# Texture analysis of muscle MRI: machine learning-based classifications in idiopathic inflammatory myopathies

**DOI:** 10.1038/s41598-021-89311-3

**Published:** 2021-05-10

**Authors:** Keita Nagawa, Masashi Suzuki, Yuuya Yamamoto, Kaiji Inoue, Eito Kozawa, Toshihide Mimura, Koichiro Nakamura, Makoto Nagata, Mamoru Niitsu

**Affiliations:** 1 Department of Radiology, Saitama Medical University Hospital, 38 Morohongo Moroyama-machi, Iruma-gun, Saitama, Japan; 2 Department of Rheumatology and Applied Immunology, Saitama Medical University Hospital, 38 Morohongo Moroyama-machi, Iruma-gun, Saitama, Japan; 3 Department of Dermatology, Saitama Medical University Hospital, 38 Morohongo Moroyama-machi, Iruma-gun, Saitama, Japan; 4 Department of Respiratory Medicine, Saitama Medical University Hospital, 38 Morohongo Moroyama-machi, Iruma-gun, Saitama, Japan

**Keywords:** Machine learning, Idiopathic inflammatory myopathies

## Abstract

To develop a machine learning (ML) model that predicts disease groups or autoantibodies in patients with idiopathic inflammatory myopathies (IIMs) using muscle MRI radiomics features. Twenty-two patients with dermatomyositis (DM), 14 with amyopathic dermatomyositis (ADM), 19 with polymyositis (PM) and 19 with non-IIM were enrolled. Using 2D manual segmentation, 93 original features as well as 93 local binary pattern (LBP) features were extracted from MRI (short-tau inversion recovery [STIR] imaging) of proximal limb muscles. To construct and compare ML models that predict disease groups using each set of features, dimensional reductions were performed using a reproducibility analysis by inter-reader and intra-reader correlation coefficients, collinearity analysis, and the sequential feature selection (SFS) algorithm. Models were created using the linear discriminant analysis (LDA), quadratic discriminant analysis (QDA), support vector machine (SVM), k-nearest neighbors (k-NN), random forest (RF) and multi-layer perceptron (MLP) classifiers, and validated using tenfold cross-validation repeated 100 times. We also investigated whether it was possible to construct models predicting autoantibody status. Our ML-based MRI radiomics models showed the potential to distinguish between PM, DM, and ADM. Models using LBP features provided better results, with macro-average AUC values of 0.767 and 0.714, accuracy of 61.2 and 61.4%, and macro-average recall of 61.9 and 59.8%, in the LDA and k-NN classifiers, respectively. In contrast, the accuracies of radiomics models distinguishing between non-IIM and IIM disease groups were low. A subgroup analysis showed that classification models for anti-Jo-1 and anti-ARS antibodies provided AUC values of 0.646–0.853 and 0.692–0.792, with accuracy of 71.5–81.0 and 65.8–78.3%, respectively. ML-based TA of muscle MRI may be used to predict disease groups or the autoantibody status in patients with IIM and is useful in non-invasive assessments of disease mechanisms.

## Introduction

Idiopathic inflammatory myopathies (IIMs) are a heterogeneous family of systemic disorders characterized by muscle weakness, muscle enzyme elevations, inflammatory changes on muscle biopsy, and extra-muscular manifestations^1,2^. The common disease groups of IIMs in adults are polymyositis (PM), dermatomyositis (DM), amyopathic dermatomyositis (ADM), and inclusion body myositis (IBM). These inflammatory myopathies show different clinical presentation patterns and responses to treatment^3–6^. Patients with PM and DM have similar therapeutic strategies involving the empirical use of corticosteroids and immunosuppressive agents^5^, whereas patients with ADM require earlier and more intensive therapy because of its poor prognosis with severe pulmonary involvement and early death^6^. Therefore, the early identification of IIM disease groups is essential for predicting clinical courses and selecting treatment plans. With the new discoveries of myositis-specific autoantibodies (MSAs) and myositis-associated autoantibodies (MAAs)^7–9^, more clinical characteristics have been obtained for IIMs. These autoantibodies are associated with distinct clinical phenotypes and may define a prognosis for a subset of patients.

In IIMs, MRI of skeletal muscles is a feasible method for assessing disease activity and identifying useful biopsy sites. Due to uniform fat suppression and no administration of contrast media, STIR MR sequences are preferred^10,11^. The proximal legs are preferentially examined because thigh muscles are mostly affected in IIM patients^12^. Although previous studies reported characteristic muscle MRI findings in IIM patients^11–16^, quantitative or semi-quantitative assessments with MRI have been limited^12^.

A texture analysis (TA) is an image analysis technique that allows for the quantification of image characteristics based on the distribution of pixels and their surface intensity or patterns^17,18^. These image characteristics are based on the microstructures of a background tissue and are sometimes imperceptible to the human visual system^17^. TA has been applied to a number of medical image assessments, including oncologic imaging^19,20^, neuroimaging^21,22^, and musculoskeletal imaging^23,24^. Recent US-based radiomics studies reported differentiation between neurogenic and myogenic diseases using musculature imaging^25^. To the best of our knowledge, an analysis of IIMs with texture features derived from muscle MRI has not yet been conducted.

The present study was performed to evaluate the diagnostic performance of ML-based MRI radiomics models for predicting disease groups in patients with IIMs. We also investigated the feasibility of classifications based on autoantibodies (e.g., anti-Jo1 and anti-ARS antibodies).

## Methods

The present study was approved by the Research Ethics Committee of Saitama Medical University Hospital as a retrospective medical imaging data analysis using TA and a deep-learning technique. The requirement for informed consent was waived by the Committee (approval number 20041.01). All experiments were performed in accordance with the relevant guidelines and regulations.

### Patients

**Figure 1:**
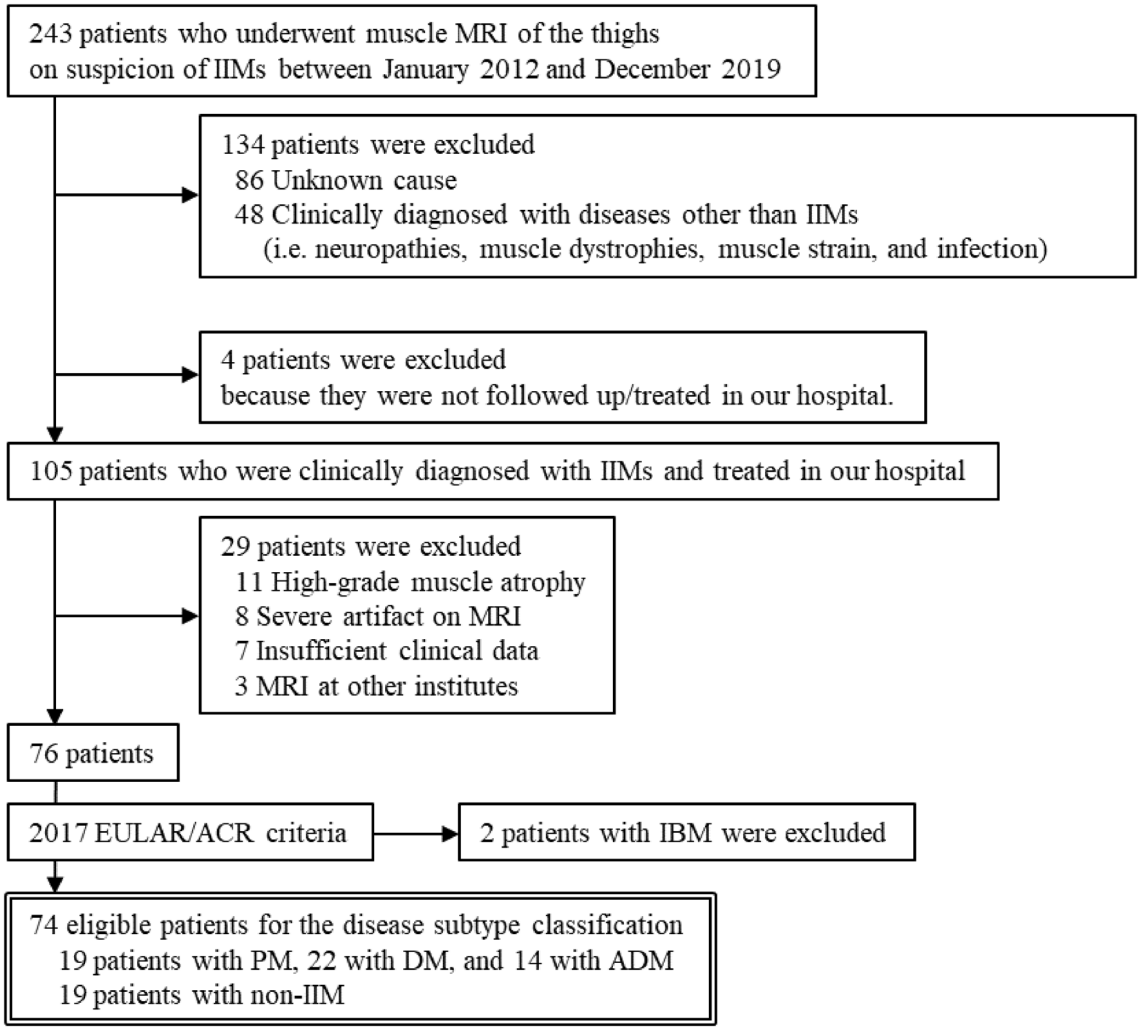
Flow chart of inclusion and exclusion criteria.

Figure 1 shows inclusion and exclusion criteria. In total, 243 patients who underwent muscle MRI of the thighs with suspicion of myositis between January 2012 and December 2019 were identified and reviewed. Exclusion criteria were as follows: 134 patients diagnosed with diseases other than myositis or an unknown cause; 4 who were not followed up nor treated after MRI in our hospital; 11 with high-grade muscle atrophy (difficulty in segmentation); 8 with severe artifacts on MRI; 7 with insufficient clinical data, and 3 who underwent MRI at other institutions. Using the 2017 European League Against Rheumatism/American College of Rheumatology (EULAR/ACR) classification criteria, the latest and most widely used criteria because of their high sensitivity and specificity^26^, the remaining 76 patients were classified into 57 with IIM (23 definite and 34 probable IIM) and 19 with non-IIM (2 possible IIM and 17 non-IIM). Fifty-seven patients with IIM were subclassified into 19 with PM, 22 with DM, 14 with ADM, and 2 with IBM. By excluding IBM (insufficient number of patients for a statistical analysis), 74 patients (19 PM, 22 DM, 14 ADM, and 19 non-IIM) were finally enrolled for the disease group classification analysis.

Patients characteristics were determined by the medical record description of their chief physician/dermatologists. We calculated aggregate scores by the definition of the 2017 EULAR/ACR. Although we classified all the patients according to the new criteria in principle, we selected patients for the IIM disease groups, particularly the ADM group, based on their clinical diagnosis because disagreement in the diagnosis of IIM disease groups has been reported in several cohort studies^27,28^.

### Data analysis procedures

A multi-class classification analysis of PM vs DM vs ADM and non-IIM vs PM vs DM vs ADM was conducted.

In a subgroup study, we investigated whether it was possible to predict the status of some representative MSAs.

**Figure 2:**
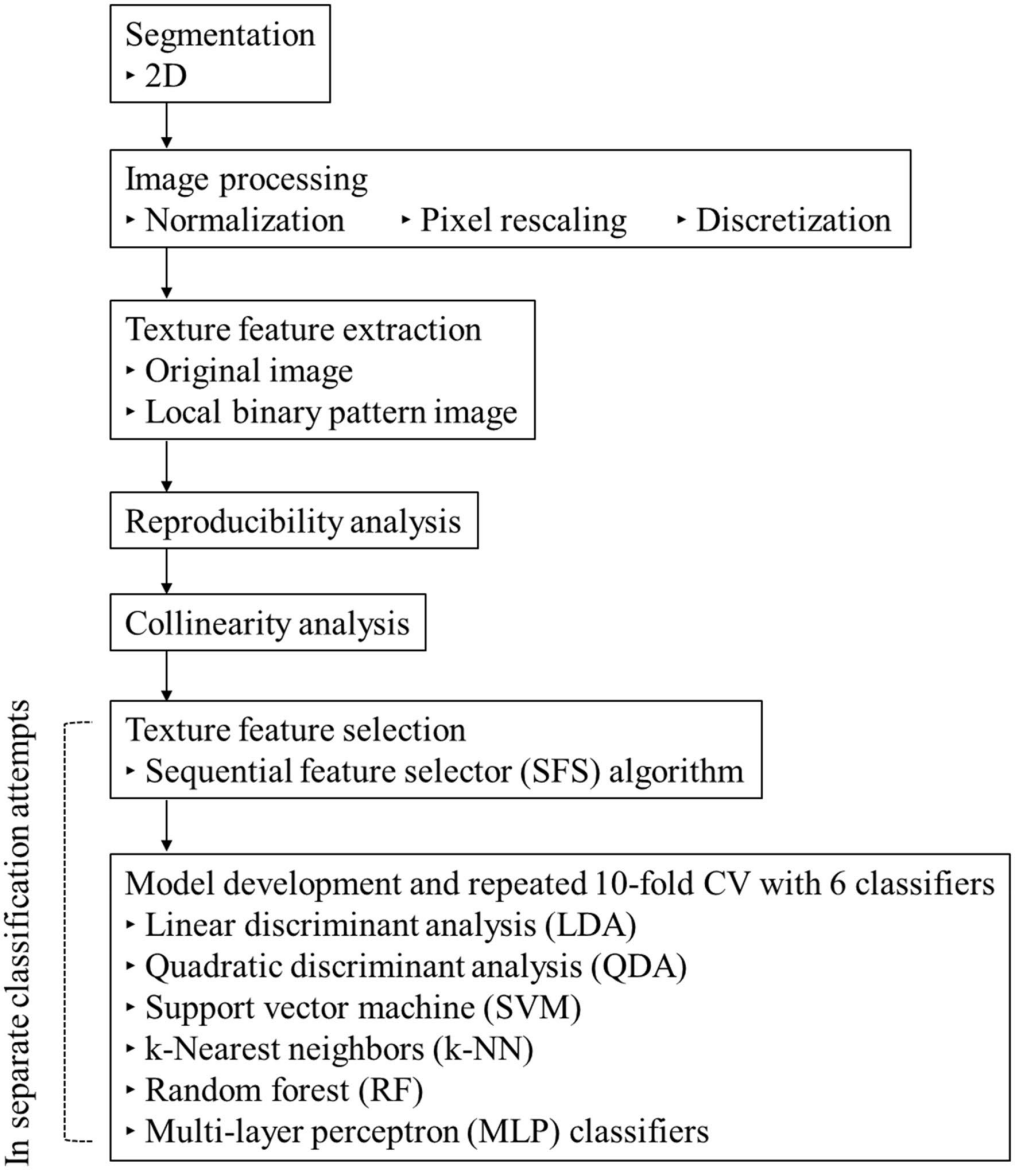
Flow chart showing the technical study pipeline. After segmentation, image processing, texture feature extraction, reproducibility analysis, and collinearity analysis were conducted in all datasets together, followed by texture feature selection and ML-based model construction in separate classification attempts. *CV* cross-validation.

The data analysis workflow is shown in Fig. 2. After segmentation, image processing, texture feature extraction, a reproducibility analysis, and collinearity analysis of all datasets together were conducted, followed by texture feature selection and ML-based model construction in separate classification attempts.

### MRI

MRI was performed using the 1.5-T system (MAGNETOM Symphony; Siemens Healthcare, Erlangen, Germany). STIR of thigh muscles in the axial plane was conducted using the following parameters: repetition time: 6500 ms; echo time: 65 ms; inversion time: 190 ms; slice thickness: 8.0 mm; flip angle: 180°; field of view: 450 × 513 mm; matrix: 307 × 384; acquisition time: 153 s.

### Segmentation

**Figure 3:**
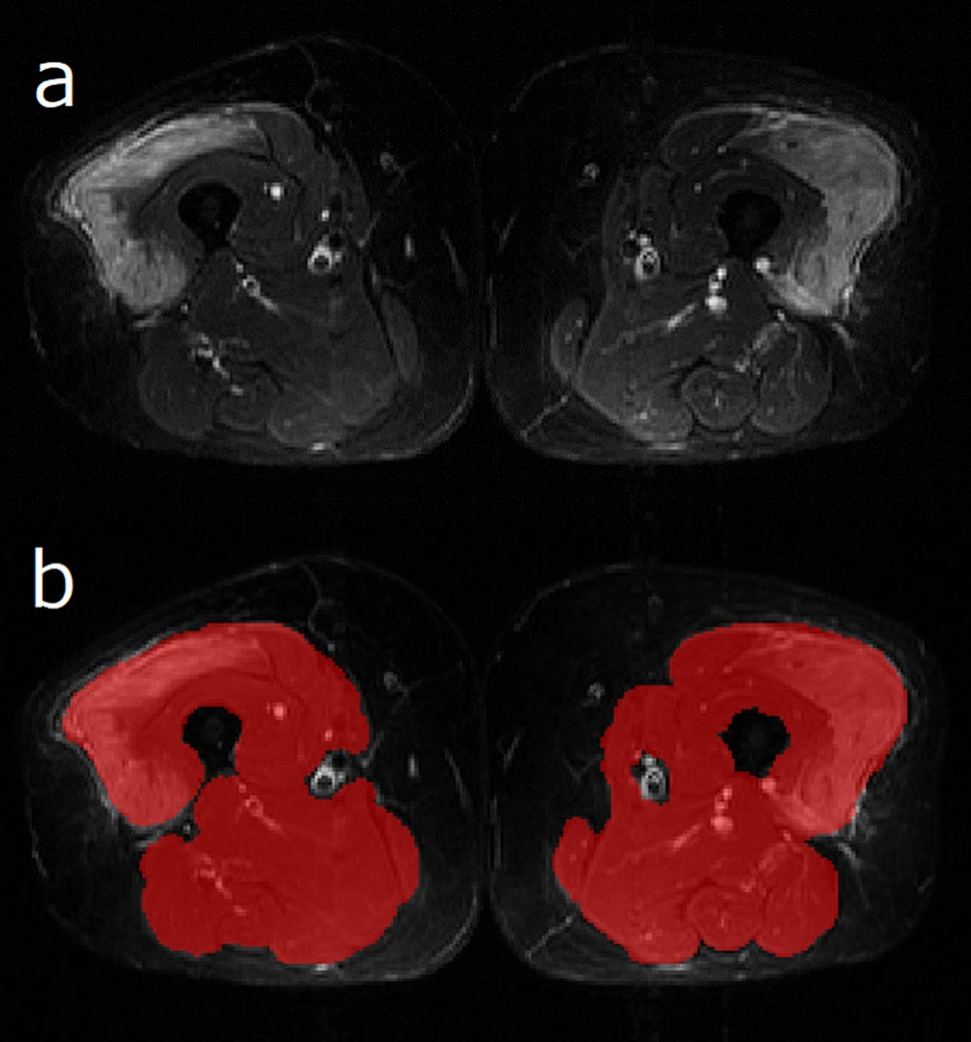
Representative segmentation style in a 67-year-old woman with PM. (**a**) An unenhanced STIR image of thigh muscles in the axial plane was examined. (**b**) The whole area of the muscles in the proximal thighs was segmented as a ROI (red shaded area), excluding the epimysium.

Muscle segmentation was performed using open-source software (ITK-SNAP version 3.8.0). A two-dimensional region of interest (ROI) that covered the whole area of one slice of a muscle MR image of the proximal thighs and excluded the epimysium was selected for each subject (see Fig. 3). Two radiologists with 20 and 4 years of experience performed the ROI delineation in an independent manner. A senior radiologist performed tumor segmentation again with a minimum interval of 2 months. Segmentation was performed on the same image slice assessed by another radiologist with 5 years of experience. All three radiologists were blinded to clinical information.

### Texture feature extraction

To avoid data heterogeneity bias, all MRI data were subjected to imaging normalization (the intensity of the image was scaled to 0–100) and resampled to the same resolution (3 × 3 × 3 mm) before feature extraction.

The calculation of texture features was performed using an open-source software package capable of extracting a large panel of engineered features from medical images (PyRadiomics version 2.1.0). Texture features were calculated based on six feature classes (first-order statistics, the gray-level co-occurrence matrix (GLCM), gray-level dependence matrix (GLDM), gray-level run-length matrix (GLRLM), gray-level size zone matrix (GLSZM), and neighboring gray-tone difference matrix (NGTDM)). Other than the 93 original features (18 first-order, 24 GLCM, 14 GLDM, 16 GLRLM, 16 GLSZM, and 5 NGTDM features), 93 filtered images using local binary pattern (LBP) were obtained and the results were compared with each other.

### Dimensional reduction of texture features

After numeric values had been normalized as z-scores, the dimensional reduction was performed in two consecutive steps: a reproducibility analysis and collinearity analysis.

To evaluate intra-observer and inter-observer reproducibilities, intraclass correlation coefficient (ICC) values were calculated for each texture feature. Features with excellent reproducibility (ICC ≥ 0.8) in intra-observer and inter-observer analyses were included in further analyses.

A collinearity analysis was conducted using Pearson’s correlation coefficient (r). The threshold for collinearity was r = 0.7. Features with high collinearity were excluded from the analysis. In the case of a feature pair having high collinearity, the one with the lowest collinearity with the other features remained in the analysis.

### Feature selection and ML–based classification

The sequential feature selection (SFS) algorithm, a wrapper-based greedy search algorithm, was used for feature selection^29^. A radiomics model was created based on a limited number of selected features (3–4 features according to the number of patients) with the lowest collinearity socores^30^. A linear discriminant analysis (LDA), quadratic discriminant analysis (QDA), support vector machine (SVM), k-nearest neighbors (k-NN), random forest (RF), and multi-layer perceptron (MLP) classifiers were used for model development, with all using default parameter settings. Ten-fold cross-validation repeated 100 times was performed for classification models. The performance of classifiers was evaluated by the area under the curve (AUC). Accuracy, sensitivity, specificity, precision, and the F-measure were calculated based on the confusion matrix of classification results.

### Statistical analysis

Statistical analyses were performed using an opensource software package (Python scikit-learn 0.22.1). Differences in patient characteristics were assessed using 2-sample *t*-tests and chi-squared tests. Values of p < 0.05 were considered to be significant.

## Results

### Clinical characteristics

The study included 74 patients (19 PM, 22 DM, 14 ADM, and 19 non-IIM). Mean age was lower in the ADM group than in the other groups. Muscle weakness was rarer and CK levels were lower in the ADM group than in the other groups. Patient characteristics are shown in Supplementary Table S1 online. Regarding the 19 non-IIM patients in the present study, 4 were clinically diagnosed with anti-ARS antibody-positive myositis, 3 with anti-SRP antibody-positive myositis, 3 with anti-mitochondrial antibody-positive myositis, 1 with systemic lupus erythematosus, 3 with systemic sclerosis, 1 with probable PM, and 4 with possible PM/DM by the Bohan and Peter criteria. Among the 19 patients with PM, 22 with DM, 14 with ADM, and 19 with non-IIM, 18, 19, 11 and 18 patients were not taking any medications, respectively. All the remaining patients, 1 with PM, 3 with DM, 3 with ADM and 1 with non-IIM, were under the maintenance dose (3–6 mg/day) of prednisolone, and they underwent MRI because of disease relapse.

### Segmentation

The ROI delineation was performed by two radiologists with 20 and 4 years of experience in an independent manner. The mean sizes of the ROIs placed by the radiologists were 484.4 (SD = 113.2) and 492.0 (SD = 123.9) cm^2^, respectively. All segmentation was performed on the 1/3 to 1/4 proximal level of the thighs.

### Dimensional reduction with the reproducibility test and collinearity analysis

Among the 93 original features, the mean ICC value was 0.926 (SD = 0.101) in the inter-observer reproducibility test and 0.975 (SD = 0.029) in the intra-observer reproducibility test. Eighty and 93 features had excellent inter-reader and intra-reader reproducibilities (ICC ≥ 0.8), respectively. The number of features with excellent reproducibility in both analyses was 80. By excluding features with high collinearity (r ≥ 0.7), the number of features was further reduced to 11. Eleven representative features and their respective ICCs are shown in Table 1, and their distribution and collinearity status are shown in Supplementary Figs. S1 and S2 online, respectively.

**Table 1:** Selected original texture features for machine learning–based classifications of IIM and non-IIM disease groups.

Code	Selected original texture features		Intraclass correlation coefficient (ICC)	
	Feature class	Feature name code	Intra-observer	Inter-observer
TexF1	First-order	Kurtosis	0.962	0.872
TexF2	First-order	Interquartile range	0.988	0.986
TexF3	First-order	Total energy	0.986	0.971
TexF4	GLCM	Cluster prominence	0.984	0.956
TexF5	GLCM	Correlation	0.983	0.953
TexF6	GLCM	Difference average	0.988	0.977
TexF7	GLCM	Imc2	0.988	0.986
TexF8	GLCM	Maximum probability	0.983	0.981
TexF9	GLDM	Large dependence high gray-level emphasis	0.980	0.909
TexF10	GLDM	Dependence non-uniformity	0.931	0.924
TexF11	NGTDM	Coarseness	0.939	0.935

*GLCM* gray-level co-occurrence matrix, *GLDM* gray-level dependence matrix, *NGTDM* neighboring gray-tone difference matrix.

On the other hand, among 93 LBP features, the mean ICC value was 0.776 (SD = 0.207) in the inter-observer reproducibility test, and 0.859 (SD = 0.100) in the intra-observer reproducibility test. Fifty-four and 60 features had excellent inter-reader and intra-reader reproducibilities (ICC ≥ 0.8), respectively. The number of features with excellent reproducibility in both analyses was 54. By excluding features with high collinearity (r ≥ 0.7), the number of features was further reduced to 9. Nine representative features and their respective ICCs are shown in Table 2, with their distribution and collinearity status in Supplementary Figs. S3 and S4 online, respectively.

**Table 2:** Selected LBP texture features for machine learning–based classifications of IIM and non-IIM disease groups.

Code	Selected LBP texture features		Intraclass correlation coefficient (ICC)	
	Feature class	Feature name code	Intra-observer	Inter-observer
TexFL1	First-order	Total energy	0.978	0.968
TexFL2	First-order	Variance	0.974	0.942
TexFL3	GLCM	Cluster shade	0.937	0.897
TexFL4	GLCM	Contrast	0.881	0.824
TexFL5	GLCM	Difference entropy	0.941	0.930
TexFL6	GLRLM	Long run emphasis	0.822	0.801
TexFL7	GLRLM	Long run low gray-level emphasis	0.890	0.803
TexFL8	GLSZM	Gray-level nonuniformity	0.919	0.896
TexFL9	NGTDM	Busyness	0.911	0.892

*LBP* local binary pattern, *GLCM* gray-level co-occurrence matrix, *GLRLM* gray-level run length matrix, *GLSZM* gray-level size zone matrix, *NGTDM* neighboring gray-tone difference matrix.

### Feature selection and ML–based multi-class classification of IIM disease groups

The SFS algorithm associated with a univariate analysis (p < 0.05) provided 3 to 4 features for each classifier (i.e. LDA, QDA, SVM, k-NN, RF, and MLP classifiers). We constructed multi-class classification models based on the selected features, and evaluated their performance via the tenfold cross-validation repeated 100 times.

The top classification score for original features was obtained in the LDA classifier: the macro-average AUC was 0.683 (SD = 0.012), with an accuracy of 58.6% (SD = 2.0%), macro-average precision of 59.1% (SD = 2.9%), and macro-average recall of 56.7% (SD = 2.1%). Equivalent classification scores were also observed in the QDA and SVM classifiers.

The best classification scores for LBP features were obtained in the LDA classifier: the macro-average AUC was 0.767 (SD = 0.011), with an accuracy of 61.2% (SD = 2.5%), macro-average precision of 61.6% (SD = 2.4%), and macro-average recall of 61.9% (SD = 2.3%). In k-NN classifiers, the macro-average AUC was 0.714 (SD = 0.015), with an accuracy of 61.4% (SD = 2.7%), macro-average precision of 67.4% (SD = 3.4%), and macro-average recall of 59.8% (SD = 2.7%).

All classification attempts for original and LBP features are summarized in Tables 3 and 4, with confusion matrices in Supplementary Figs. S5 and S6 and roc curves in Figs. S7 and S8 online, respectively.

**Table 3:** Performance of multi-class classifications of IIM groups (original features).

	Accuracy	Recall	Precision	AUC
	(%)	(%)	(%)	
**LDA (TexF1 + TexF3 + TexF4 + TexF6)**				
Macro-average	58.6 ± 2.0	56.7 ± 2.1	59.1 ± 2.9	0.683 ± 0.012
PM		52.1 ± 4.8	52.1 ± 2.9	0.605 ± 0.021
DM		72.5 ± 1.1	61.5 ± 1.8	0.684 ± 0.013
ADM		45.6 ± 4.0	63.7 ± 6.9	0.721 ± 0.018
**QDA (TexF1 + TexF8 + TexF10)**				
Macro-average	57.4 ± 3.0	56.2 ± 2.3	59.7 ± 2.3	0.668 ± 0.016
PM		62.2 ± 3.7	50.4 ± 3.0	0.626 ± 0.021
DM		61.0 ± 4.6	60.5 ± 3.7	0.648 ± 0.028
ADM		45.4 ± 3.4	68.0 ± 3.8	0.688 ± 0.027
**SVM (TexF1 + TexF3 + TexF4)**				
Macro-average	58.1 ± 2.0	56.2 ± 2.5	56.9 ± 2.5	0.629 ± 0.030
PM		45.8 ± 6.0	52.2 ± 3.6	0.505 ± 0.053
DM		71.9 ± 2.0	65.6 ± 2.8	0.656 ± 0.035
ADM		44.1 ± 7.3	52.7 ± 6.1	0.688 ± 0.041
**k-NN (TexF1 + TexF3 + TexF10)**				
Macro-average	55.5 ± 2.5	53.5 ± 2.6	66.5 ± 3.5	0.649 ± 0.019
PM		66.4 ± 4.6	45.1 ± 2.8	0.575 ± 0.028
DM		58.9 ± 3.9	59.6 ± 3.5	0.662 ± 0.023
ADM		35.4 ± 4.9	94.8 ± 9.1	0.705 ± 0.027
**RF (TexF1 + TexF2 + TexF7 + TexF11)**				
Macro-average	47.2 ± 3.2	45.4 ± 3.1	47.3 ± 3.8	0.642 ± 0.022
PM		49.1 ± 7.2	41.1 ± 4.3	0.612 ± 0.035
DM		55.8 ± 5.5	53.6 ± 3.4	0.648 ± 0.031
ADM		31.2 ± 4.7	47.2 ± 8.4	0.651 ± 0.035
**MLP (TexF1 + TexF3 + TexF6)**				
Macro-average	54.6 ± 2.1	53.5 ± 2.0	56.6 ± 2.4	0.647 ± 0.016
PM		43.5 ± 3.5	43.5 ± 3.3	0.517 ± 0.035
DM		67.2 ± 4.1	57.6 ± 3.3	0.657 ± 0.020
ADM		49.9 ± 4.3	68.6 ± 6.5	0.726 ± 0.020

Data are means ± standard deviations. Feature name codes are as follows: TexF1 = kurtosis, TexF2 = interquartile range, TexF3 = total energy, TexF4 = cluster prominence, TexF5 = correlation, TexF6 = difference average, TexF7 = imc2, TexF8 = maximum probability, TexF9 = large dependence high gray-level emphasis, TexF10 = dependence non-uniformity, TexF11 = coarseness.

*LDA* linear discriminant analysis, *QDA* quadratic discriminant analysis, *SVM* support vector machine, *k-NN* k-nearest neighbors classifier, *RF* random forest classifier, *MLP* multi-layer perceptron.

**Table 4:** Performance of multi-class classifications of IIM groups (LBP features).

	Accuracy	Recall	Precision	AUC
	(%)	(%)	(%)	
**LDA (TexFL2 + TexFL5 + TexFL7 + TexFL8)**				
Macro-average	61.2 ± 2.5	61.9 ± 2.3	61.6 ± 2.4	0.767 ± 0.011
PM		55.0 ± 5.6	56.4 ± 3.5	0.693 ± 0.016
DM		61.8 ± 3.6	62.2 ± 3.3	0.756 ± 0.015
ADM		68.8 ± 4.0	66.1 ± 3.3	0.815 ± 0.014
**QDA (TexFL1 + TexFL2 + TexFL3)**				
Macro-average	55.1 ± 3.1	55.8 ± 5.5	54.9 ± 5.6	0.724 ± 0.016
PM		43.7 ± 6.3	52.7 ± 4.7	0.695 ± 0.030
DM		58.2 ± 4.2	64.8 ± 4.7	0.685 ± 0.018
ADM		65.5 ± 6.7	47.3 ± 3.4	0.755 ± 0.022
**SVM (TexFL1 + TexFL2 + TexFL7)**				
Macro-average	54.7 ± 3.9	54.9 ± 3.9	55.6 ± 4.0	0.717 ± 0.020
PM		45.8 ± 6.0	46.0 ± 5.4	0.621 ± 0.033
DM		59.5 ± 5.7	57.3 ± 4.5	0.692 ± 0.030
ADM		59.5 ± 6.8	63.6 ± 6.4	0.801 ± 0.016
**k-NN (TexFL1 + TexFL6 + TexFL7)**				
Macro-average	61.4 ± 2.7	59.8 ± 2.7	67.4 ± 3.4	0.714 ± 0.015
PM		68.9 ± 6.1	57.3 ± 4.0	0.718 ± 0.027
DM		65.2 ± 4.3	58.0 ± 3.1	0.685 ± 0.024
ADM		45.1 ± 4.4	86.9 ± 8.7	0.740 ± 0.013
**RF (TexFL2 + TexFL3 + TexFL6 + TexFL8)**				
Macro-average	52.6 ± 4.0	52.3 ± 3.9	54.4 ± 4.6	0.735 ± 0.022
PM		51.4 ± 6.9	54.0 ± 5.7	0.668 ± 0.036
DM		55.2 ± 6.2	48.3 ± 3.9	0.649 ± 0.032
ADM		50.2 ± 6.3	61.0 ± 9.1	0.853 ± 0.023
**MLP (TexFL1 + TexFL2 + TexFL7)**				
Macro-average	51.1 ± 3.2	51.9 ± 3.0	52.0 ± 3.2	0.740 ± 0.012
PM		47.3 ± 5.6	44.0 ± 4.0	0.668 ± 0.021
DM		49.4 ± 4.9	52.9 ± 4.2	0.700 ± 0.018
ADM		59.2 ± 3.7	59.1 ± 4.2	0.818 ± 0.014

*Note* Data are means ± standard deviations. Feature name codes are as follows: TexFL1 = total energy, TexFL2 = variance, TexFL3 = cluster shade, TexFL4 = contrast, TexFL5 = difference entropy, TexFL6 = long run emphasis, TexFL7 = long run low gray-level emphasis, TexFL8 = gray-level non-uniformity, TexFL9 = busyness.

*LDA* linear discriminant analysis, *QDA* quadratic discriminant analysis, *SVM* support vector machine, *k-NN* k-nearest neighbors classifier, *RF* random forest classifier, *MLP* multi-layer perceptron.

### ML-based multi-class classification attempts for non-IIM and IIM disease groups

In the multi-class classification analysis of non-IIM vs PM vs DM vs ADM, we selected representative features using the SFS algorithm associated with a univariate analysis, and evaluated their performance via the tenfold cross-validation repeated 100 times.

The classification scores for the original and LBP features were low in all representative classifiers.

The highest classification scores for original features were obtained in the LDA classifier: the macro-average AUC was 0.627 (SD = 0.013), with an accuracy of 42.7% (SD = 2.6%), macro-average precision of 40.3% (SD = 3.5%), and macro-average recall of 41.2% (SD = 2.6%). In the MLP classifier, the macro-average AUC was 0.628 (SD = 0.012), with an accuracy of 42.6% (SD = 2.7%), macro-average precision of 42.8% (SD = 3.2%), and macro-average recall of 40.9% (SD = 2.6%). On the other hand, the highest classification scores for LBP features were obtained in the RF classifier: the macro-average AUC was 0.657 (SD = 0.016), with an accuracy of 43.2% (SD = 3.3%), macro-average precision of 45.6% (SD = 4.1%), and macro-average recall of 42.5% (SD = 3.3%). In the LDA classifier, the macro-average AUC was 0.618 (SD = 0.013), with an accuracy of 41.3% (SD = 2.5%), macro-average precision of 41.5% (SD = 2.8%), and macro-average recall of 40.2% (SD = 2.4%).

All of the classification attempts for original and LBP features are summarized in Supplementary Tables S2 and S3, with confusion matrices in Supplementary Figs. S9 and S10 and roc curves in Figs. S11 and S12 online, respectively.

### Subgroup analysis of ML-based classification of representative autoantibodies

We searched our patients for the anti-Jo-1 and anti-ARS antibodies, and selected 45 patients with sufficient data for these two autoantibodies. Although we also investigated other MSA/MAAs, few patients had sufficient data (the information on representative MSA/MAAs in all patients for this analysis is shown in Supplementary Table S4 online). Therefore, in this subgroup analysis, we focused on the anti-Jo-1 and anti-ARS antibodies, and attempted to construct binary classification models for each antibody. We only applied original features because of the limited number of subjects. We selected 3 representative features using the SFS algorithm associated with a univariate analysis, and evaluated their performance by tenfold cross-validation repeated 100 times.

The classification scores for two autoantibodies were moderate to good. AUC values were 0.646–0.853 and 0.692–0.792, with accuracies of 71.5–81.0 and 65.8–78.3%, sensitivities of 25.8–62.2 and 68.3–75.6%, and specificities of 87.1–96.5 and 62.0–81.5% for the anti-Jo-1 and anti-ARS antibodies, respectively. All classification attempts for these two antibodies are summarized in Tables 5 and 6, with confusion matrices in Supplementary Figs. S13 and S14 and roc curves in Figs. S15 and S16 online, respectively.

**Table 5:** Performance of machine learning–based classifications of anti-Jo-1 antibodies.

	Accuracy	Sensitivity	Specificity	F-measure	AUC
	(%)	(%)	(%)		
LDA (TexF2 + TexF9 + TexF11)	71.5 ± 2.8	25.8 ± 5.5	90.1 ± 2.8	0.343 ± 0.066	0.646 ± 0.019
QDA (TexF2 + TexF7 + TexF11)	78.4 ± 1.7	33.9 ± 5.0	96.5 ± 1.0	0.474 ± 0.055	0.824 ± 0.027
SVM (TexF3 + TexF6 + TexF11)	79.6 ± 1.2	45.5 ± 2.8	93.5 ± 1.3	0.563 ± 0.029	0.672 ± 0.048
k-NN (TexF2 + TexF9 + TexF11)	79.9 ± 1.6	62.2 ± 4.8	87.1 ± 1.4	0.640 ± 0.034	0.845 ± 0.015
RF (TexF2 + TexF5 + TexF10)	81.0 ± 2.7	51.4 ± 7.4	93.0 ± 2.5	0.608 ± 0.065	0.853 ± 0.027
MLP (TexF4 + TexF10 + TexF11)	77.2 ± 2.5	45.2 ± 5.5	90.2 ± 2.6	0.533 ± 0.053	0.807 ± 0.023

*Note* Data are means ± standard deviations. Feature name codes are as follows: TexF1 = kurtosis, TexF2 = interquartile range, TexF3 = total energy, TexF4 = cluster prominence, TexF5 = correlation, TexF6 = difference average, TexF7 = imc2, TexF8 = maximum probability, TexF9 = large dependence high gray-level emphasis, TexF10 = dependence non-uniformity, TexF11 = coarseness.

*LDA* linear discriminant analysis, *QDA* quadratic discriminant analysis, *SVM* support vector machine, *k-NN* k-nearest neighbors classifier, *RF* random forest classifier, *MLP* multi-layer perceptron.

**Table 6:** Performance of machine learning–based classifications of anti-ARS-antibodies.

	Accuracy	Sensitivity	Specificity	F-measure	AUC
	(%)	(%)	(%)		
LDA (TexF3 + TexF6 + TexF10)	78.3 ± 2.2	75.5 ± 3.3	81.5 ± 2.5	0.815 ± 0.025	0.792 ± 0.018
QDA (TexF3 + TexF5 + TexF10)	71.1 ± 2.9	68.3 ± 4.7	74.3 ± 3.2	0.715 ± 0.033	0.731 ± 0.030
SVM (TexF4 + TexF7 + TexF10)	76.4 ± 2.2	74.5 ± 4.0	78.7 ± 2.6	0.771 ± 0.025	0.752 ± 0.015
k-NN (TexF4 + TexF8 + TexF10)	73.2 ± 2.6	75.6 ± 2.6	70.5 ± 5.1	0.751 ± 0.022	0.742 ± 0.019
RF (TexF3 + TexF4 + TexF10)	65.8 ± 3.5	69.1 ± 5.2	62.0 ± 4.2	0.682 ± 0.037	0.692 ± 0.022
MLP (TexF4 + TexF9 + TexF10)	68.9 ± 3.9	72.6 ± 5.1	64.7 ± 6.4	0.713 ± 0.037	0.693 ± 0.029

*Note* Data are means ± standard deviations. Feature name codes are as follows: TexF1 = kurtosis, TexF2 = interquartile range, TexF3 = total energy, TexF4 = cluster prominence, TexF5 = correlation, TexF6 = difference average, TexF7 = imc2, TexF8 = maximum probability, TexF9 = large dependence high gray-level emphasis, TexF10 = dependence non-uniformity, TexF11 = coarseness.

*LDA* linear discriminant analysis, *QDA* quadratic discriminant analysis, *SVM* support vector machine, *k-NN* k-nearest neighbors classifier, *RF* random forest classifier, *MLP* multi-layer perceptron.

## Discussion

In the present study, we found that ML-based TA of muscle MRI has the potential to distinguish between PM, DM, and ADM. In contrast, ML models distinguishing between non-IIM and IIM disease groups had low classification accuracy. We also showed that our ML models have the potential to predict the status of anti-Jo-1 and anti-ARS antibodies. Since IIMs are rare disorders, we were unable to collect a large number of IIM patients. Therefore, this analysis is a small-scale proof-of-concept study that demonstrates the potential of MR-based TA to predict disease groups or the autoantibody status.

To the best of our knowledge, the potential value of MR-based TA for discriminating IIM disease groups has not yet been assessed. Apart from TA, attempts have been made to differentiate between IIM disease subtypes using conventional MRI findings. Previous studies demonstrated that a subcutaneous high signal intensity (HSI), fascial HSI, and the patchy or diffuse distribution of HSI in muscle are useful MRI findings for differentiating between PM and DM^11–16^. Ukichi et al. assessed the likelihood of DM using a scoring system with several characteristic MRI findings^16^. Although classification performance in the present study was lower, several points need to be considered that emphasize the advantages of our models. We built a multi-class classification model for PM, DM and ADM, which is more practical for clinical applications. Furthermore, instead of using conventional morphological parameters that are subject to individual interpretation and inter-observer variability, we introduced radiomics imaging analyses that extract various quantitative features from medical images and overcome these issues. In addition, we further developed classification models for autoantibodies, which are useful for clinical practice in recent antibody-oriented medicine.

IIMs are now diagnosed based on the findings of clinical and histopathological examinations. Although muscle and skin biopsies are widely accepted methods for defining the diagnosis of IIMs, they are invasive and susceptible to significant sampling bias. In previous studies, false-negative results were reported in 10–20% of all IIM muscle biopsies due to sampling errors caused by the scattered distribution of focal disease activity^31–34^. The 2017 EULAR/ACR criteria were recently introduced, which permit diagnoses using a two-version scoring system with and without muscle biopsy^26^. Although the new criteria have the advantages of high diagnostic performance and flexibility, disagreements in the diagnosis of IIM disease groups have been reported in several cohort studies^27,28^. MRI, which is not incorporated into the new criteria, is not invasive and has the potential to characterize IIM disease subtypes. A quantitative radiomics assessment of muscle MRI, as shown in our approach, may be a more objective and feasible method for IIM diagnoses and disease subtype classifications.

In the present study, several complex TA features were valuable for differentiating between IIM disease groups; GLCM features describes the second-order statistical information of gray levels between neighboring pixels in an image^35^; the LBP-2D filtered-feature represents a comparison of center pixels and their surrounding pixels. Since these complex TA features have been suggested to reflect underlying pathomorphological texture patterns in various fields of medical imaging^36–38^, the explanation of TA features provided in the present study needs to be complemented by further evidence, including histopathology.

Conventional ML classifiers, such as LDA, SVM, k-NN, and RF, were mainly examined in the present study instead of using a deep-learning or convolutional neural network (CNN) approach. Since deep-learning is now widely used for image classification to facilitate the diagnosis of various diseases, it would add values and expect improvement in classification rates to introduce the deep-learning or CNN method. Multi-task deep CNN models were recently applied to the diagnosis of neurodiseases and achieved high classification performance^39^. These models are suited to our theme because complex multi-omics data are particularly important in IIM or other collagen diseases, and a deep CNN approach will assist in the construction of favorable classification models for these diseases. As a preliminary study on CNN-based classification models, we implemented the MLP classifier, which is the simplest form of an artificial neural network. In the present study, the MLP classifier provided similar or slightly lower results than the other conventional classifiers. Since MLP is considered be a favorable estimator in non-linear models, our models may be approximated to linear models rather than complex non-linear models. However, since this is a small-scale study, different results may have been obtained if we employed large samples as well as independent training and test cohorts.

Overall, our radiomics models distinguished between IIM disease groups with moderate diagnostic accuracy, but with poor accuracy between non-IIM and IIM disease groups. It is important to note that even patients with non-IIM decided by the 2017 ACR/EULAR criteria may have IIM to some degree because the criteria are in themselves a prediction model using an aggregate scoring system derived from several variables. According to its definitions, “possible IIM” and “non-IIM”, which were combined as non-IIM in the present study, correspond to a possibility of ≥ 50% and < 55%, and < 50%, respectively^26^.

In a recent study, among 111 patients who were diagnosed with IIM clinically, 89 (80.2%) were classified as having probable/definite IIM using the 2017 ACR/EULAR criteria, while the other 22 (19.8%) were in the false-negative possible IIM/non-IIM group^28^. In the present study, all 19 patients with non-IIM were clinically diagnosed with IIM. Except for two patients with ADM (confirmed by skin biopsy), the other 17 were treated as PM; however, it was not clear whether at least 4 patients had PM or DM. Moreover, 12 out of the 19 non-IIM patients showed HSI on muscle STIR MRI.

Sampling errors may occur with biopsies, which is consistent with previous findings, and this reduced the likelihood of a diagnosis of IIM within the classification criteria because of increases in aggregate score cut points with the addition of biopsy information. Other reported diagnostic factors that may lead to false classification results in the IIM/non-IIM group included the autoantibody status and skin manifestations^27,28^. Due to the uncertainty and heterogeneity of the non-IIM group decided by the 2017 EULAR/ACR criteria, it appears to be more important to include appropriate control groups, such as normal or other disease groups, rather than a non-IIM group or to construct autoantibody-oriented classification models if the goal is to construct useful classification models for clinical practice. Since it is not currently possible to include new samples, this is a subject for future analyses.

The present results also provide a promising perspective on the classification of autoantibodies. We achieved good diagnostic performance using radiomics models for the anti-Jo1 and anti-ARS antibodies. Anti-Jo1 antibodies are the most common autoantibodies among IIM (up to 20% of IIM)^40^. They are included as anti-ARS antibodies, which define the clinical phenotype called anti-synthetase syndrome (ASS), including myositis, interstitial lung disease, arthritis, Raynaud’s phenomenon, and mechanics hands^41^. Previous studies reported that characteristic histopathological features and muscle MRI patterns in active ASS^42,43^. In the present study, we speculate that a high magnitude of voxel values and inhomogeneity in an image, which corresponded to the features such as total energy, cluster prominence, dependence non-uniformity and coarseness, may be characteristics of ASS; however, this needs to be evaluated in future studies that include histopathology. Regarding other autoantibodies, Pinal-Fernandez et al. showed that anti-SRP-positive immune-mediated necrotizing myopathy (IMNM) had more severe atrophy and fatty replacement than anti-HMGCR-positive IMNM^44^. Due to our small sample size, we were unable to construct classification models for several other autoantibodies. However, based on the importance of autoantibodies in IIMs, we need to evaluate multi-class classification models of several important autoantibodies in future studies.

The present study had limitations. The number of patients examined was not sufficient to construct a ML-based classification model. The rarity of these disorders prevented the collection of a large sample size. To avoid overfitting, we performed several feature reduction steps, including a collinearity analysis and SFS algorithms, and constructed a model with a limited number of features and multiple classifiers. We also performed a cross-validation of the calculated models to avoid overestimation. Nevertheless, future studies with a large sample size and independent training and test cohorts will provide supportive evidence for the diagnostic value of our radiomics models. Another limitation is that the present study lacked appropriate control groups, such as normal or other disease groups, as described above. Moreover, it is of greater clinical importance to construct a comprehensive classification model in consideration of clinical, serological, and pathological data. We had to correlate between histopathology and MRI TA values, and also we had to compare our radiomics models with human readers. The further development of our models will be achieved by addressing these issues in the future. In addition, our analysis only considered the intra-muscular area. Based on previous findings, the inclusion of the extra-muscular area, particularly the subcutaneous area, may provide more accurate results. Similarly, we only used STIR images in our radiomics analysis. We did not include additional TA on contrast-enhanced images because contrast-enhanced sequences were not available in all patients. A recent study on characteristic MRI findings of IIM stated that contrast-enhanced sequences were useful for the differentiation of disease groups, whereas STIR images provided similar results and were beneficial considering the risk and cost of contrast media.

In conclusion, ML-based TA of muscle MRI has potential as a method for predicting disease groups or autoantibody status in patients with IIM. With further studies to verify its reproducibility and viability, TA may become a clinically feasible technique that will be of assistance in non-invasive assessments of underlying disease mechanisms and help guide therapeutic decisions.

## Supplementary Information

Supplementary Information.

## Data Availability

The datasets generated during and/or analyzed during the current study are available from the corresponding author on reasonable request.

## Abbreviations

ADM: Amyopathic dermatomyositis
ASS: Anti-synthetase syndrome
AUC: Area under the curve
DM: Dermatomyositis
GLCM: Gray-level co-occurrence matrix
GLDM: Gray-level dependence matrix
GLRLM: Gray-level run-length matrix
GLZLM: Gray-level zone length matrix
HSI: High signal intensity
IBM: Inclusion body myositis
ICC: Intraclass correlation coefficient
IIM: Idiopathic inflammatory myopathy
k-NN: K-nearest neighbors
LBP: Local binary pattern
LDA: Linear discriminant analysis
QDA: Quadratic discriminant analysis
MAA: Myositis-associated autoantibody
ML: Machine learning
MLP: Multi-layer perceptron
MRI: Magnetic resonance imaging
MSA: Myositis-specific autoantibody
NGTDM: Neighboring gray-tone difference matrix
PM: Polymyositis
RF: Random forest
ROI: Region of interest
STIR: Short-tau inversion recovery
SVM: Support vector machine
TA: Texture analysis
