# Supplementary material for: Texture analysis of muscle MRI: machine learning-based classifications in idiopathic inflammatory myopathies
Source: Sci Rep. 2021 May 10;11:9821. doi: 10.1038/s41598-021-89311-3 (PMC8110584; doi:10.1038/s41598-021-89311-3)

Title of the Article:

Texture Analysis of Muscle MRI: Machine Learning-based Classifications of Idiopathic Inflammatory Myopathies

Names of Authors:

Keita Nagawa^1^, Masashi Suzuki^1^, Yuuya Yamamoto^1^, Kaiji Inoue^1^, Eito Kozawa^1^, Toshihide Mimura^2^, Koichiro Nakamura^3^, Makoto Nagata^4^, Mamoru Niitsu^1^

Affiliations and Addresses of the Authors:

1. Department of Radiology, Saitama Medical University Hospital, 38 Morohongo Moroyama-machi, Iruma-gun, Saitama, Japan

2. Department of Rheumatology and Applied Immunology, Saitama Medical University Hospital, 38 Morohongo Moroyama-machi, Iruma-gun, Saitama, Japan

3. Department of Dermatology, Saitama Medical University Hospital, 38 Morohongo Moroyama-machi, Iruma-gun, Saitama, Japan

4. Department of Respiratory Medicine, Saitama Medical University Hospital, 38 Morohongo Moroyama-machi, Iruma-gun, Saitama, Japan


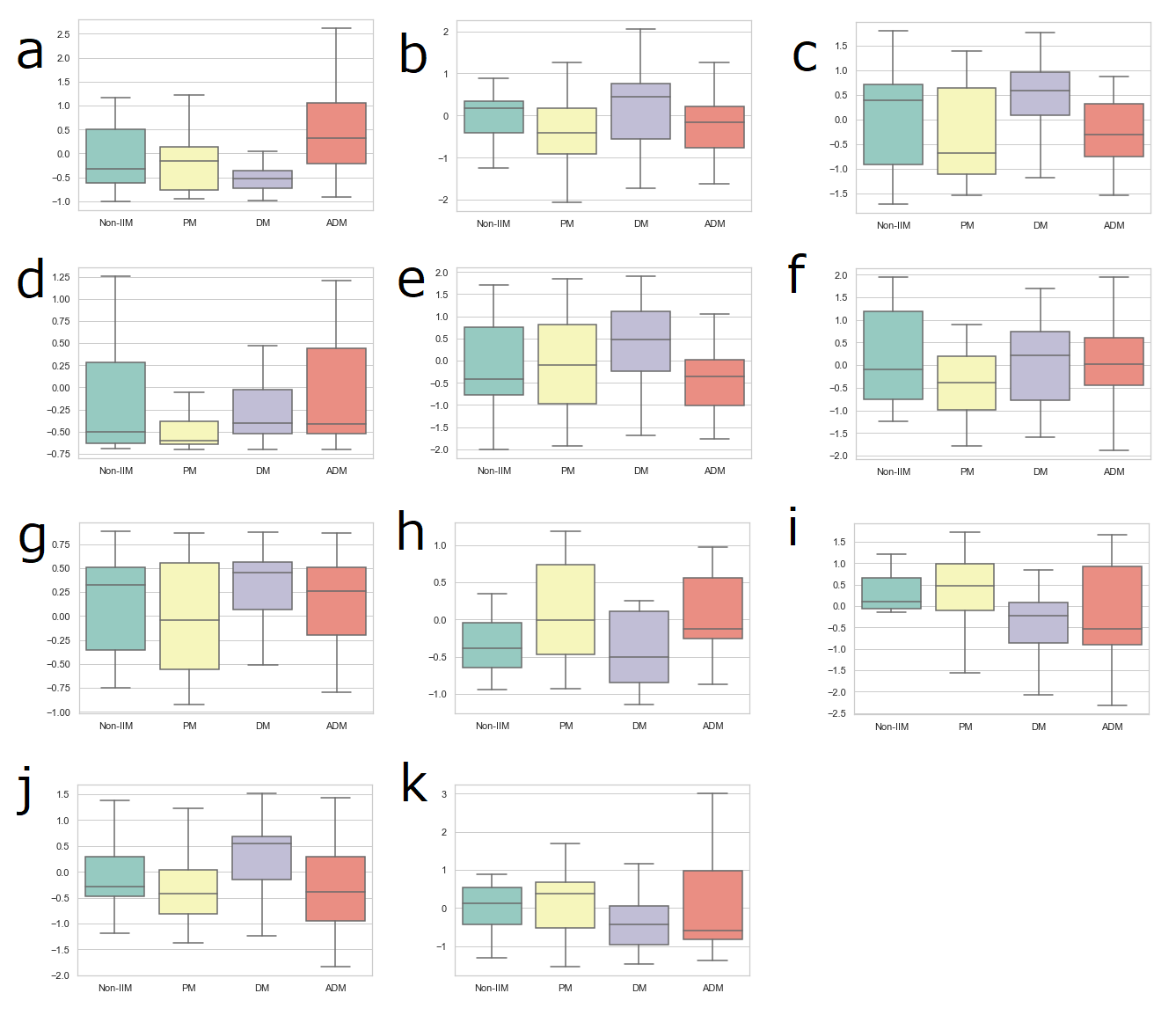


Supplementary Figure S1. Box plots show the distribution of 11 selected original features as normalized texture parameter values of each IIM and non-IIM group (with non-IIM denoted by a green box, PM by a yellow box, DM by a gray box, and ADM by a red box).


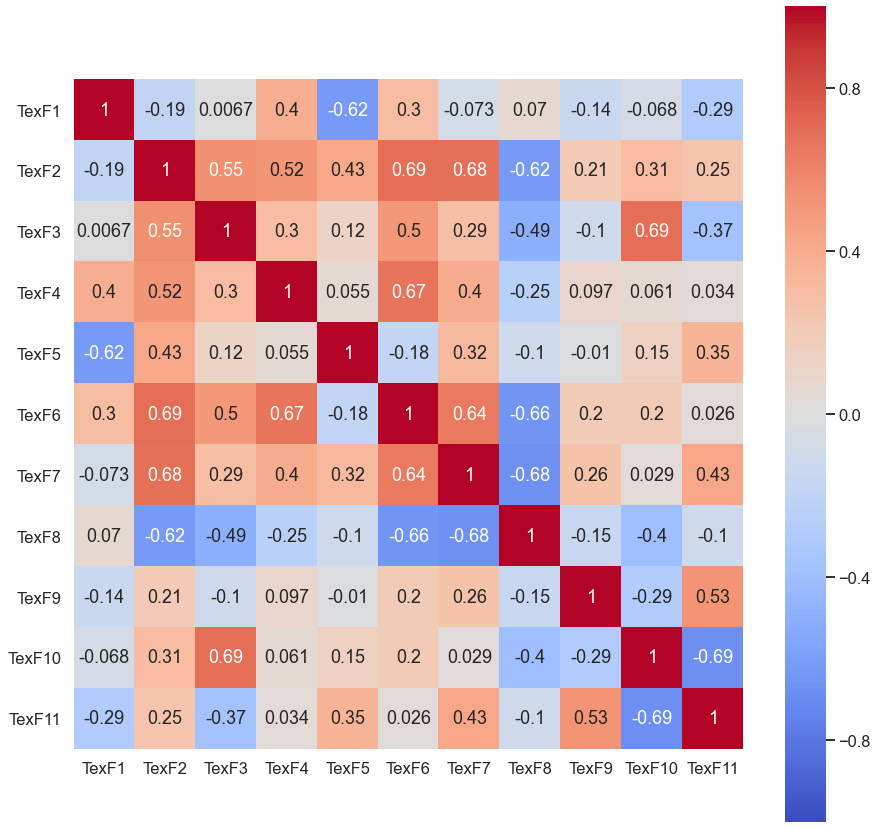
 Supplementary Figure S2. A correlation matrix shows the collinearity status of 11 selected original features. A cross-correlation was not observed (r ≥0.7) among the selected features.


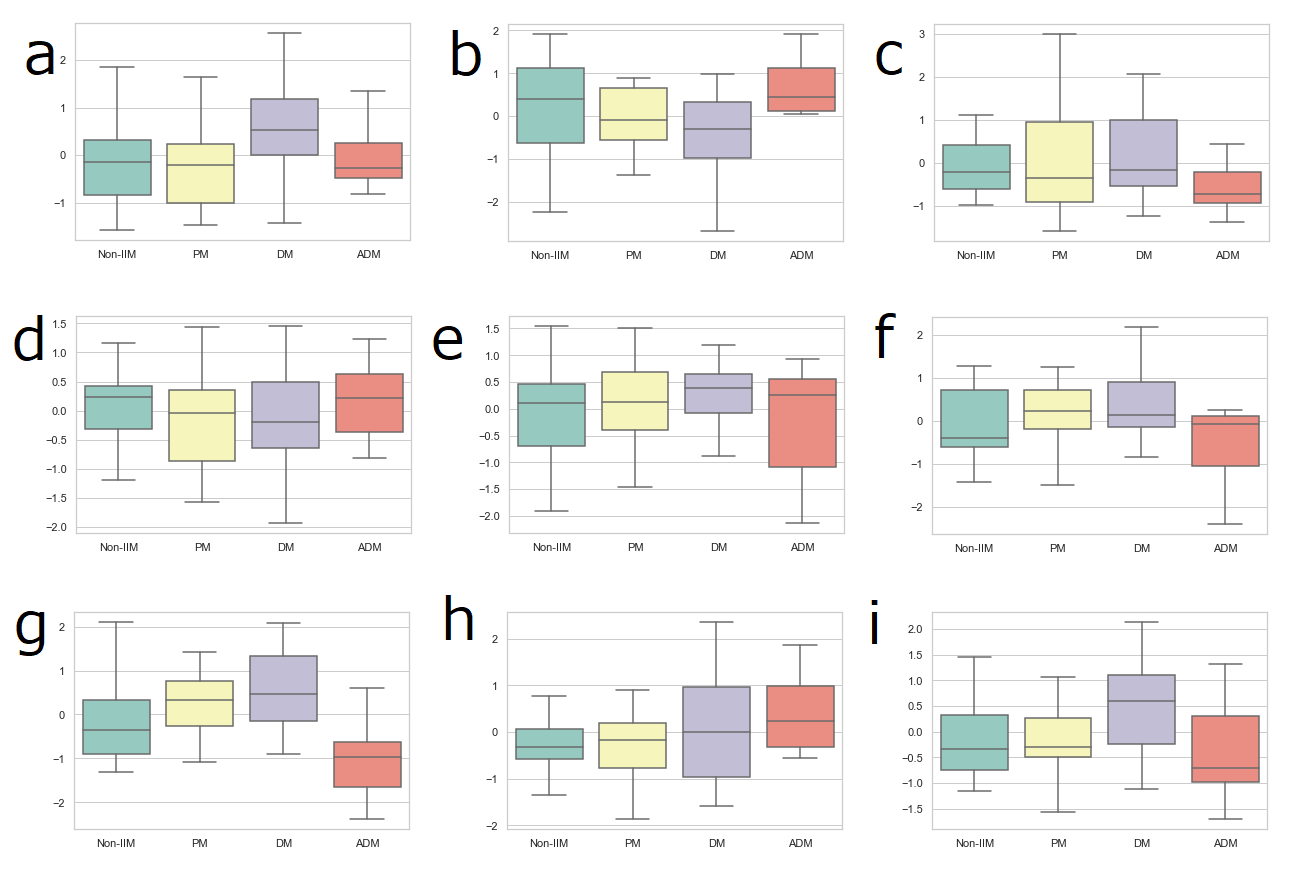
Supplementary Figure S3. Box plots show the distribution of 9 selected local binary pattern (LBP) features as normalized texture parameter values of each IIM and non-IIM group (with non-IIM denoted by a green box, PM by a yellow box, DM by a gray box, and ADM by a red box). The name codes of the selected LBP features are as follows: TexFL1 = total energy (**a**), TexFL2 = variance (**b**), TexFL3 = cluster shade (**c**), TexFL4 = contrast (**d**), TexFL5 = difference entropy (**e**), TexFL6 = long run emphasis (**f**), TexFL7 = long run low gray-level emphasis (**g**), TexFL8 = gray-level non-uniformity (**h**), TexFL9 = busyness (**i**).


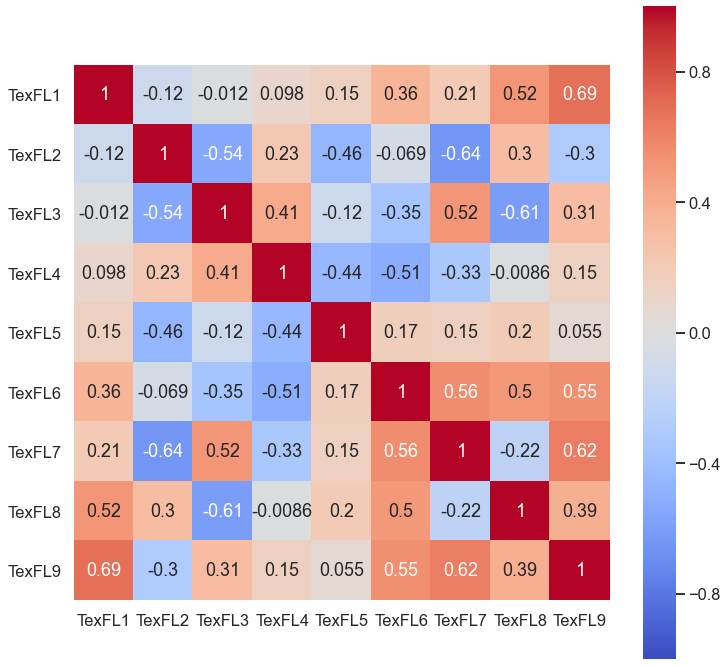


Supplementary Figure S4. A correlation matrix shows the collinearity status of 9 selected local binary pattern (LBP) features. A cross-correlation was not observed (r ≥0.7) among the selected features. Feature name codes are as follows: TexFL1 = total energy, TexFL2 = variance, TexFL3 = cluster shade, TexFL4 = contrast, TexFL5 = difference entropy, TexFL6 = long run emphasis, TexFL7 = long run low gray-level emphasis, TexFL8 = gray-level non-uniformity, TexFL9 = busyness.


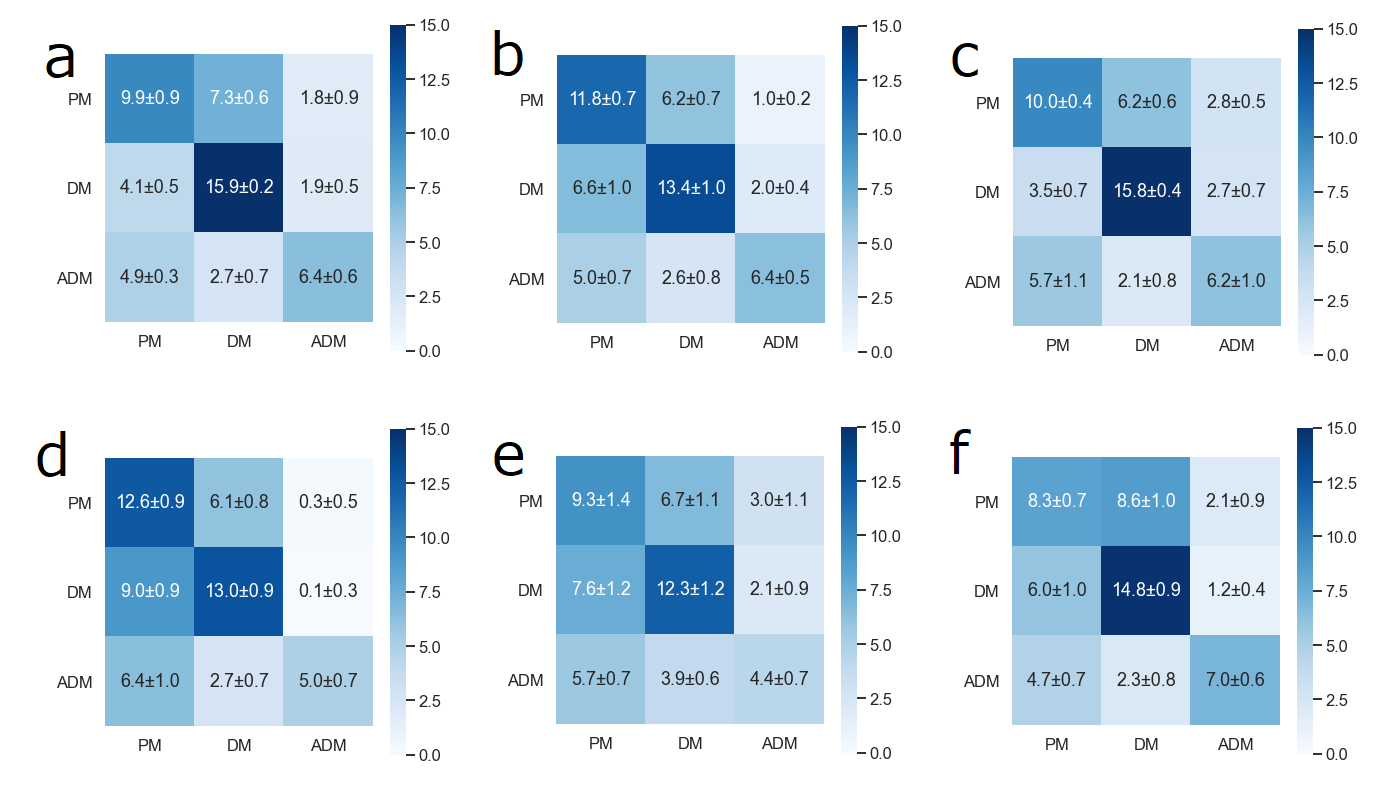


Supplementary Figure S5. Confusion matrices show the status of multi-class classifications using original features for PM vs DM vs ADM groups with a linear discriminant analysis (LDA) classifier (**a**), quadratic discriminant analysis (QDA) classifier (**b**), support vector machine (SVM) classifier (**c**), k-nearest neighbors (k-NN) classifier (**d**), random forest (RF) classifier (**e**), and multi-layer perceptron (MLP) classifier (**f**). Data are means ± standard deviations.


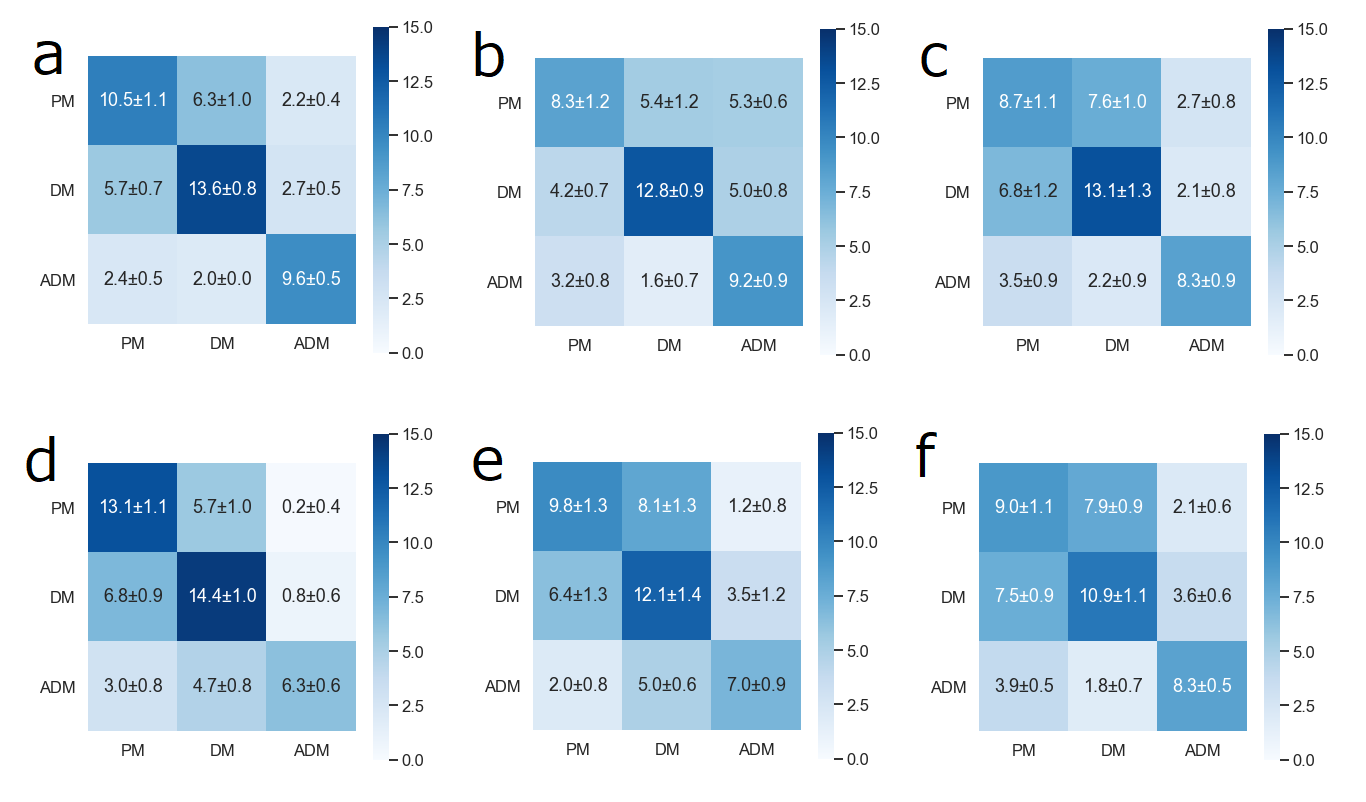


Supplementary Figure S6. Confusion matrices show the status of multi-class classification using local binary pattern (LBP) features for PM vs DM vs ADM groups with a linear discriminant analysis (LDA) classifier (**a**), quadratic discriminant analysis (QDA) classifier (**b**), support vector machine (SVM) classifier (**c**), k-nearest neighbors (k-NN) classifier (**d**), random forest (RF) classifier (**e**), and multi-layer perceptron (MLP) classifier (**f**). Data are means ± standard deviations.


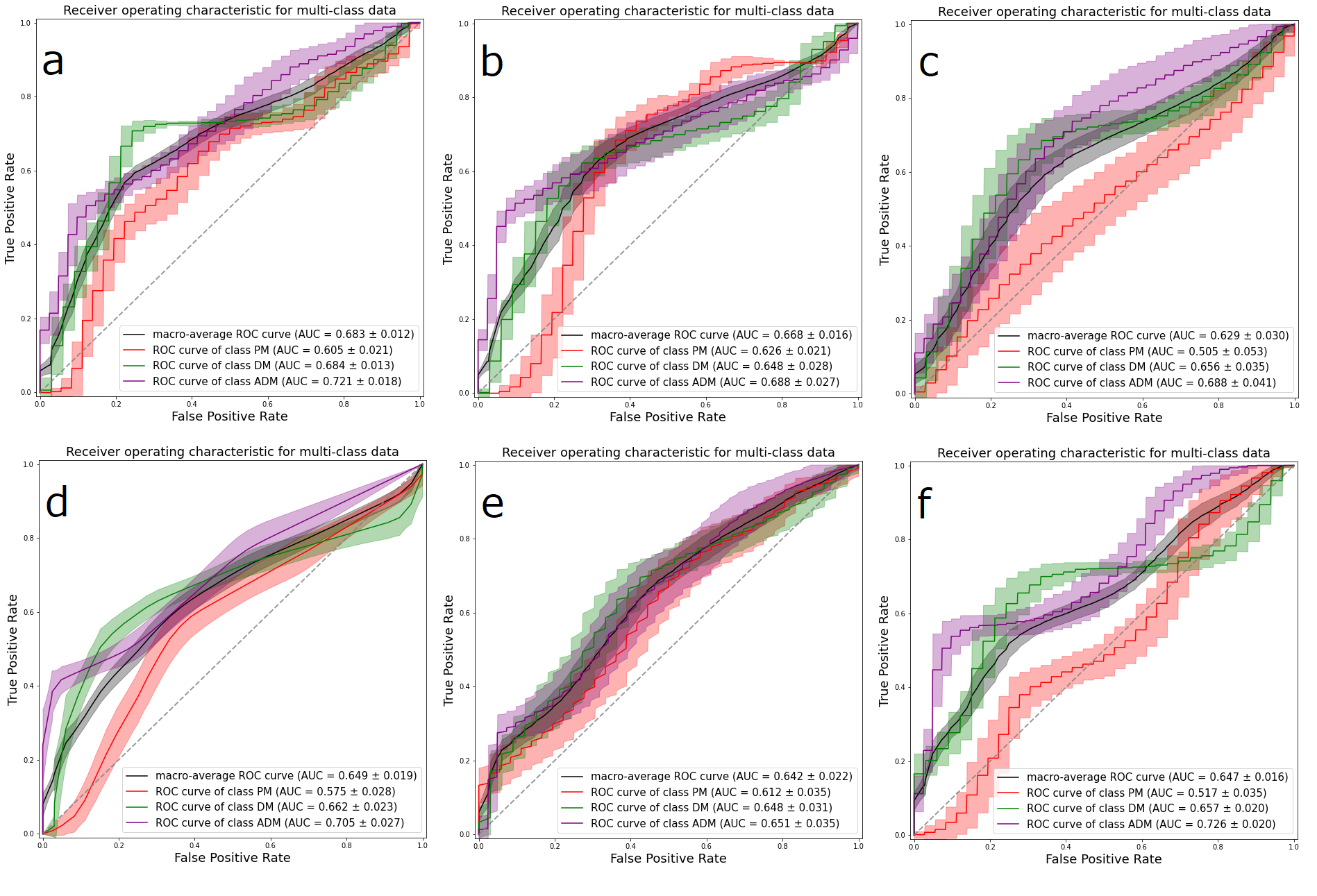


Supplementary Figure S7. ROC curves and AUC values of multi-class classifications using original features for PM vs DM vs ADM groups with a linear discriminant analysis (LDA) classifier (**a**), quadratic discriminant analysis (QDA) classifier (**b**), support vector machine (SVM) classifier (**c**), k-nearest neighbors (k-NN) classifier (**d**), random forest (RF) classifier (**e**), and multi-layer perceptron (MLP) classifier (**f**). AUCs are means ± standard deviations.


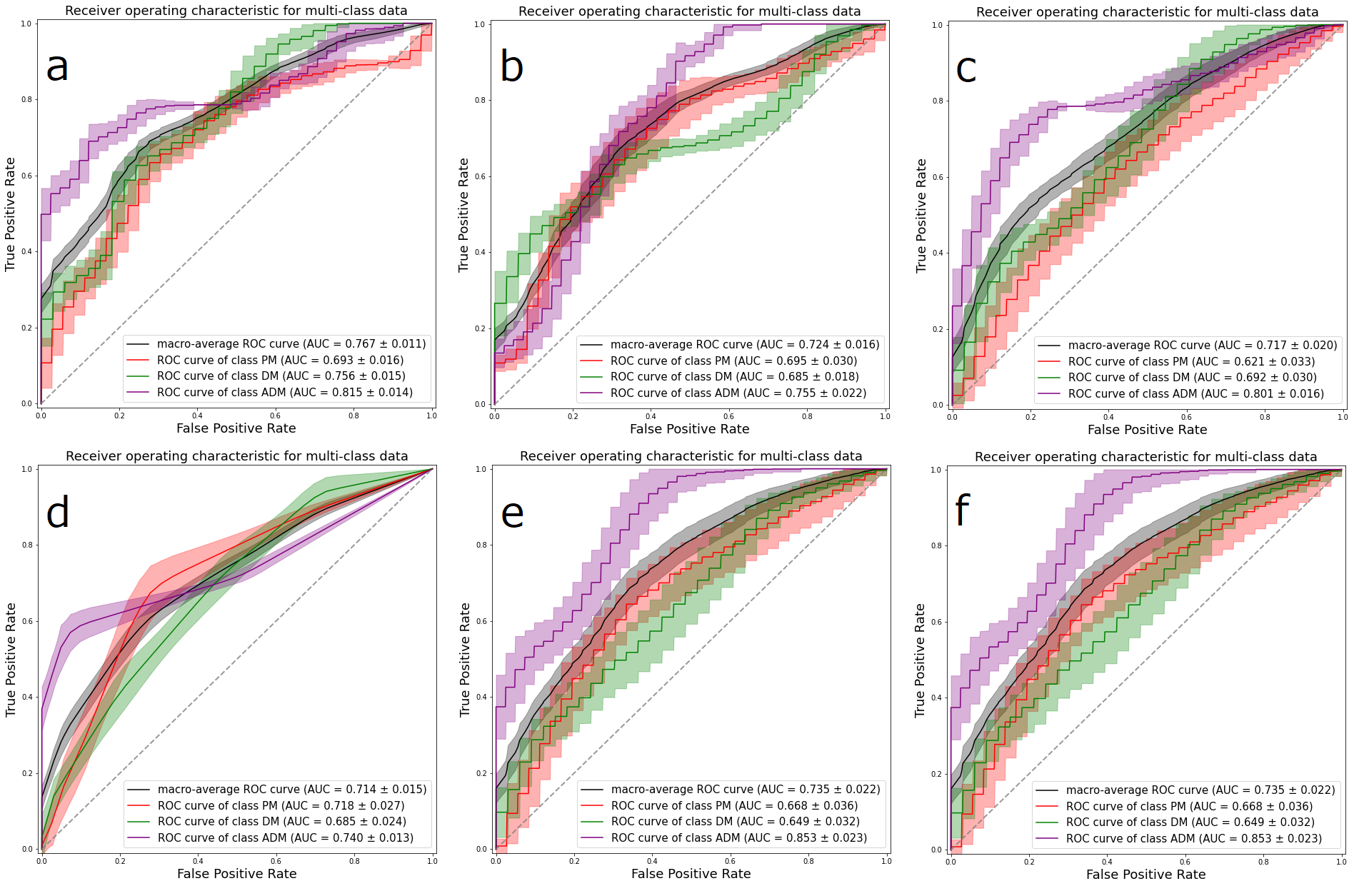


Supplementary Figure S8. ROC curves and AUC values of multi-class classifications using local binary pattern (LBP) features for PM vs DM vs ADM groups with a linear discriminant analysis (LDA) classifier (**a**), quadratic discriminant analysis (QDA) classifier (**b**), support vector machine (SVM) classifier (**c**), k-nearest neighbors (k-NN) classifier (**d**), random forest (RF) classifier (**e**), and multi-layer perceptron (MLP) classifier (**f**). AUCs are means ± standard deviations.


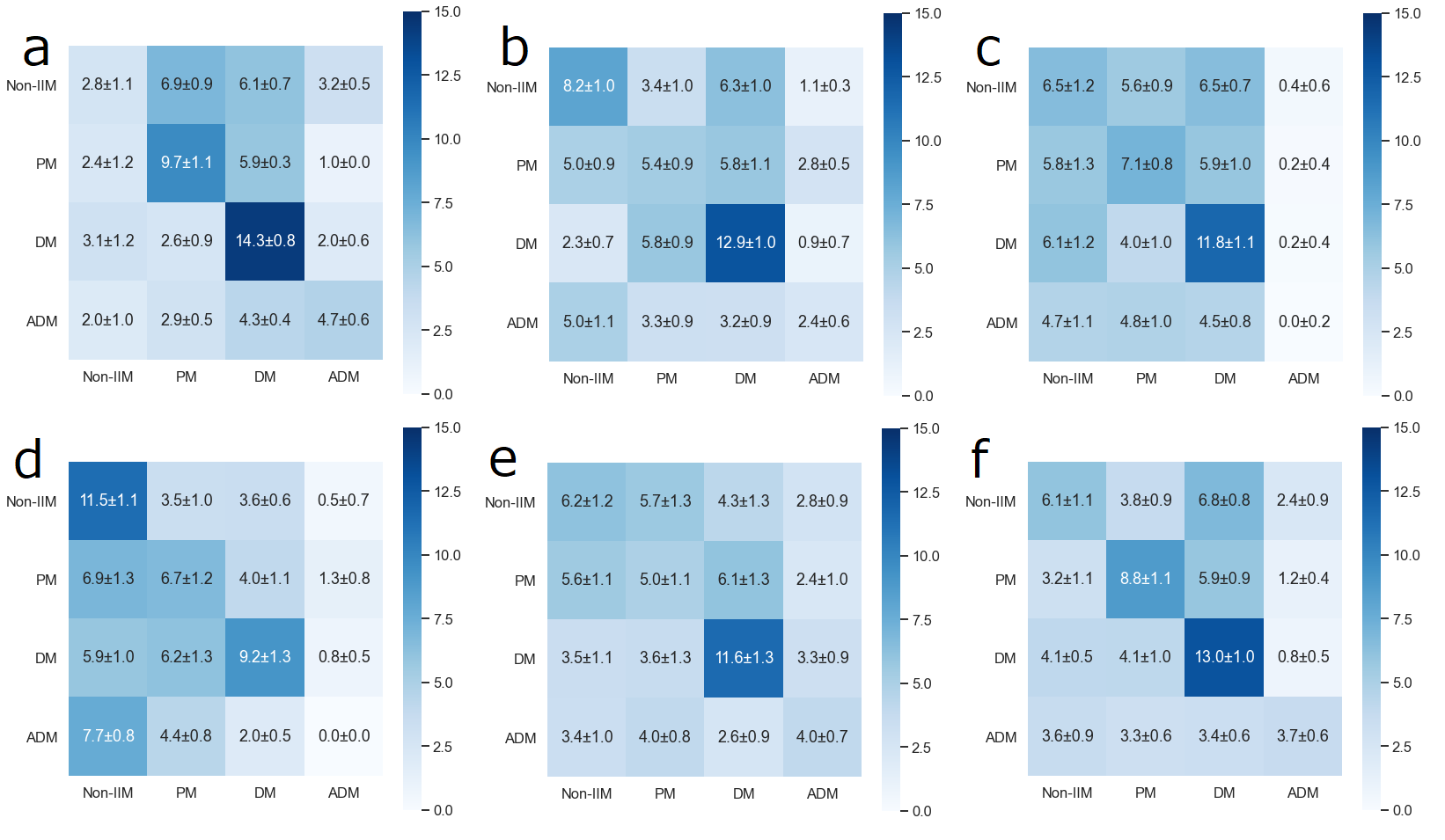


Supplementary Figure S9. Confusion matrices show the status of multi-class classification using original features for non-IIM vs PM vs DM vs ADM groups with a linear discriminant analysis (LDA) classifier (**a**), quadratic discriminant analysis (QDA) classifier (**b**), support vector machine (SVM) classifier (**c**), k-nearest neighbors (k-NN) classifier (**d**), random forest (RF) classifier (**e**), and multi-layer perceptron (MLP) classifier (**f**). Data are means ± standard deviations.


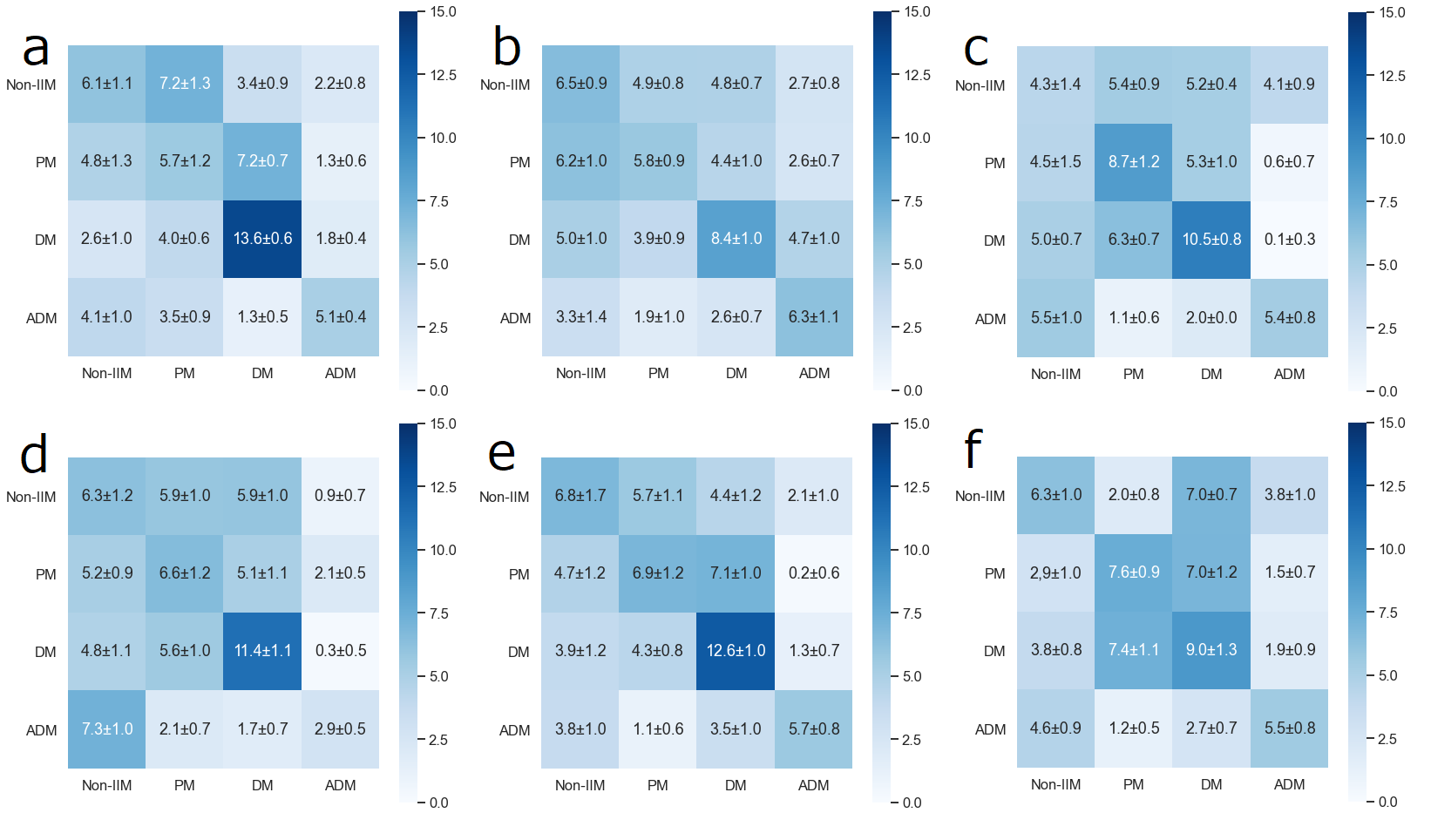


Supplementary Figure S10. Confusion matrices show the status of multi-class classification using local binary pattern (LBP) features for non-IIM vs PM vs DM vs ADM groups with a linear discriminant analysis (LDA) classifier (**a**), quadratic discriminant analysis (QDA) classifier (**b**), support vector machine (SVM) classifier (**c**), k-nearest neighbors (k-NN) classifier (**d**), random forest (RF) classifier (**e**), and multi-layer perceptron (MLP) classifier (**f**). Data are means ± standard deviations.


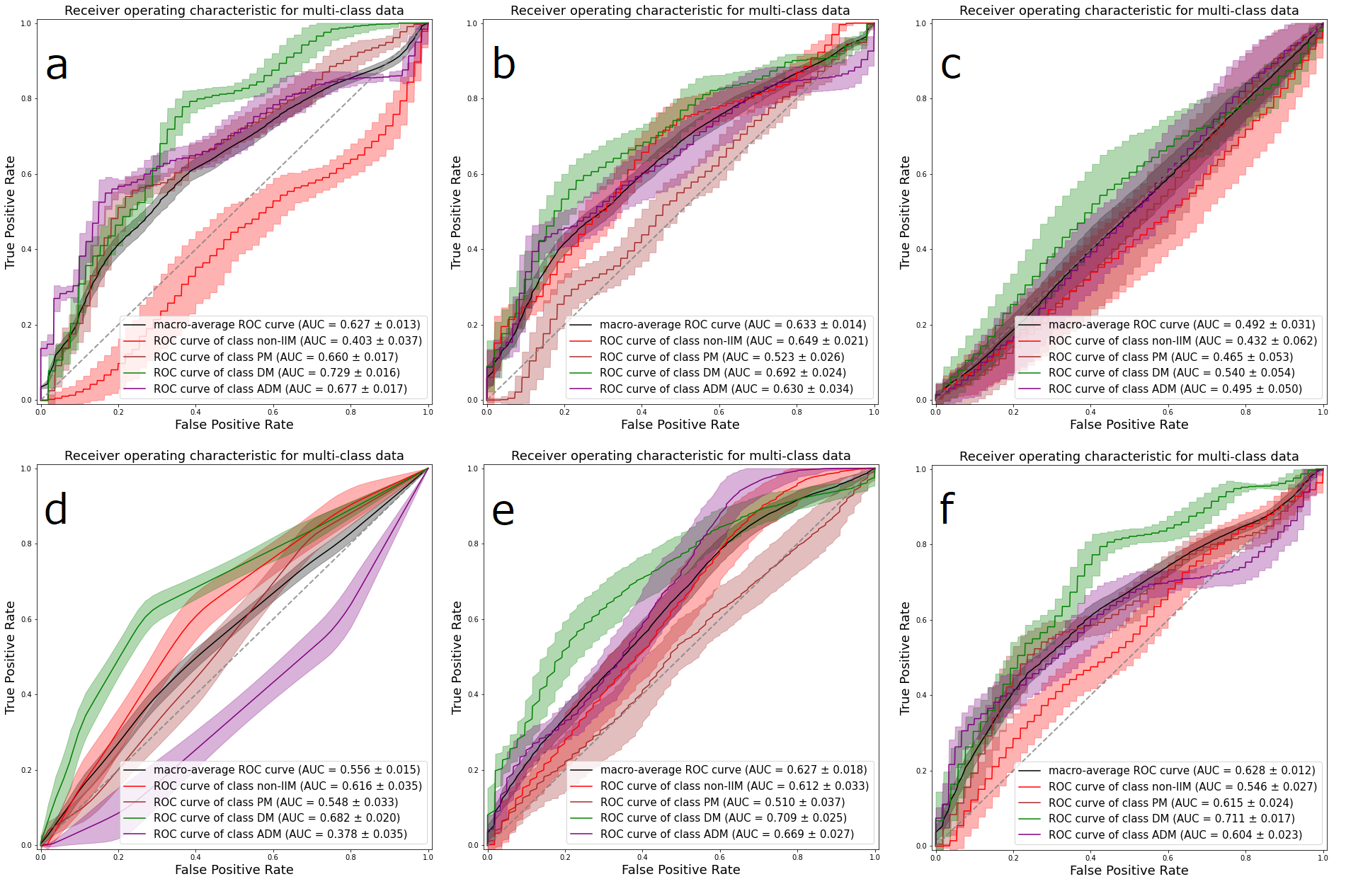


Supplementary Figure S11. ROC curves and AUC values of multi-class classifications using original features for non-IIM vs PM vs DM vs ADM groups with a linear discriminant analysis (LDA) classifier (**a**), quadratic discriminant analysis (QDA) classifier (**b**), support vector machine (SVM) classifier (**c**), k-nearest neighbors (k-NN) classifier (**d**), random forest (RF) classifier (**e**), and multi-layer perceptron (MLP) classifier (**f**). AUCs are means ± standard deviations.


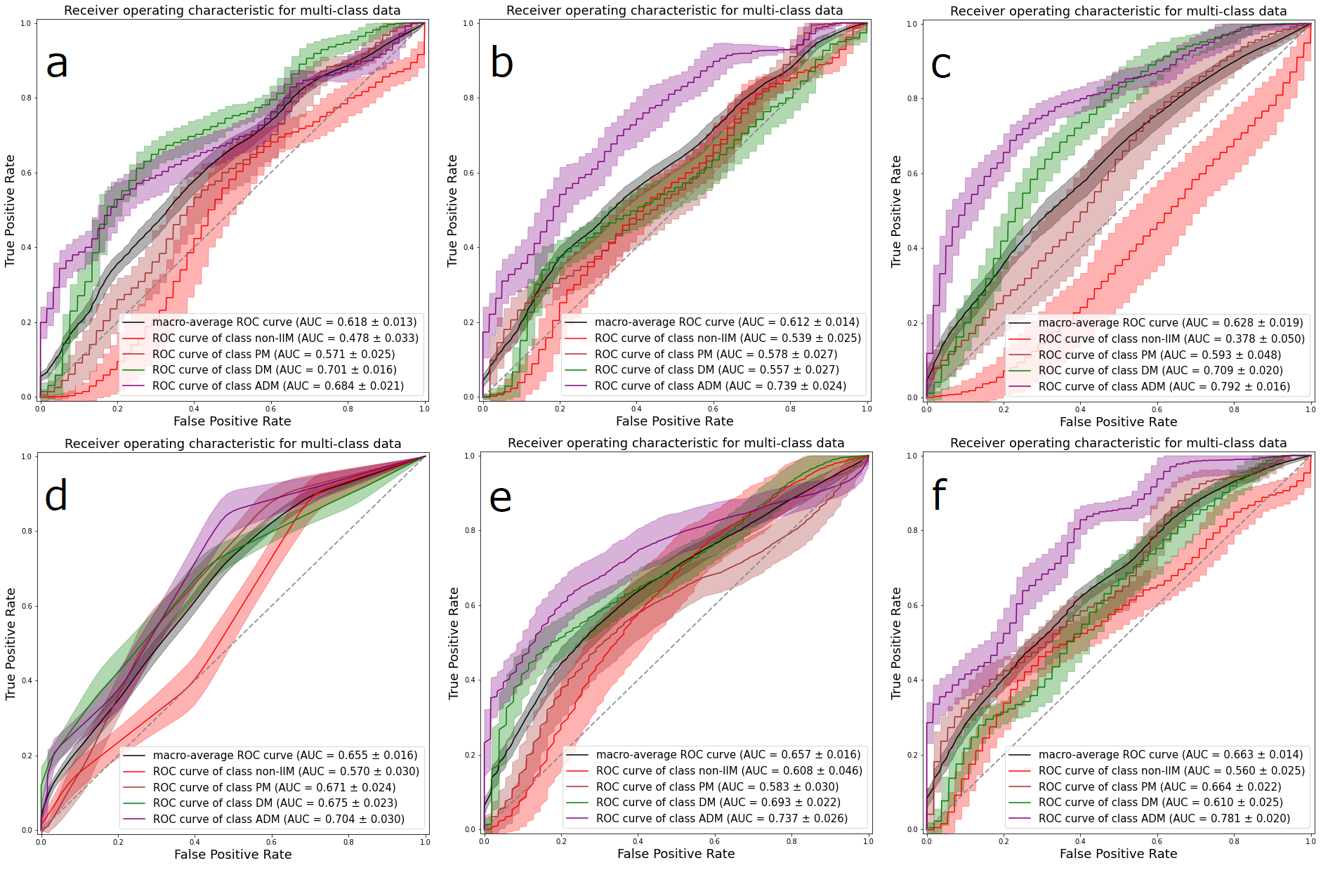


Supplementary Figure S12. ROC curves and AUC values of multi-class classifications using local binary pattern (LBP) features for non-IIM vs PM vs DM vs ADM groups with a linear discriminant analysis (LDA) classifier (**a**), quadratic discriminant analysis (QDA) classifier (**b**), support vector machine (SVM) classifier (**c**), k-nearest neighbors (k-NN) classifier (**d**), random forest (RF) classifier (**e**), and multi-layer perceptron (MLP) classifier (**f**). AUCs are means ± standard deviations.


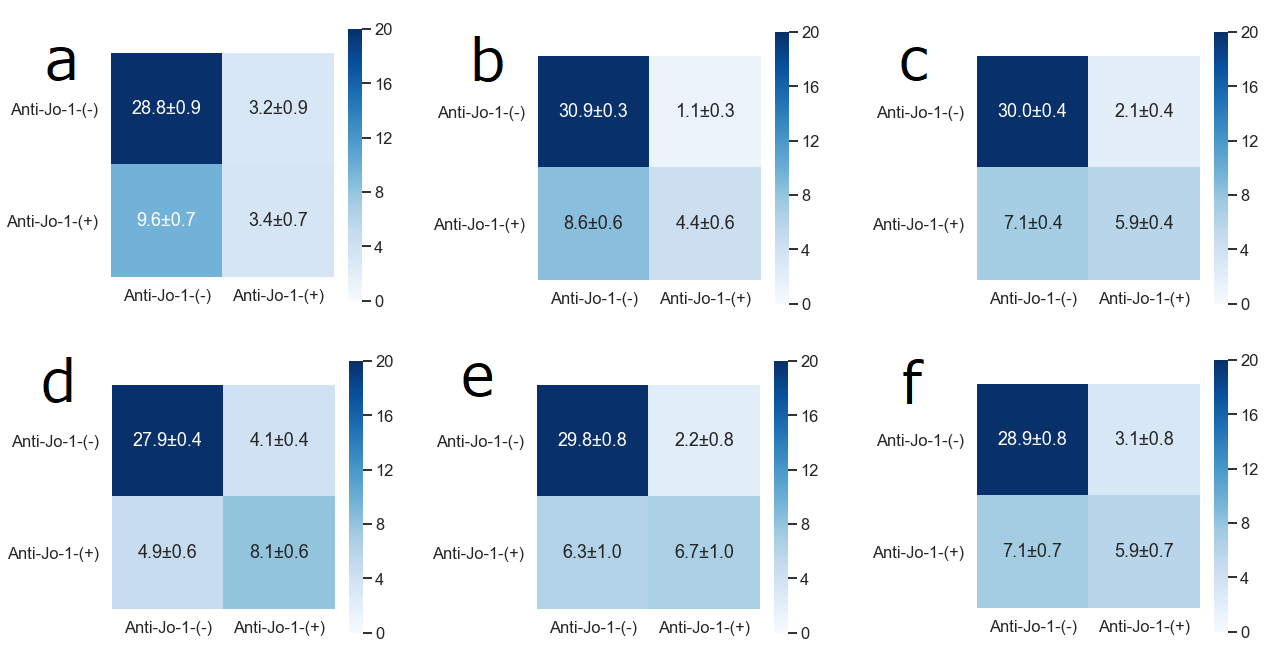


Supplementary Figure S13. Confusion matrices show the status of binary classifications for anti-Jo-1 autoantibodies with a linear discriminant analysis (LDA) classifier (**a**), quadratic discriminant analysis (QDA) classifier (**b**), support vector machine (SVM) classifier (**c**), k-nearest neighbors (k-NN) classifier (**d**), random forest (RF) classifier (**e**), and multi-layer perceptron (MLP) classifier (**f**). Data are means ± standard deviations.


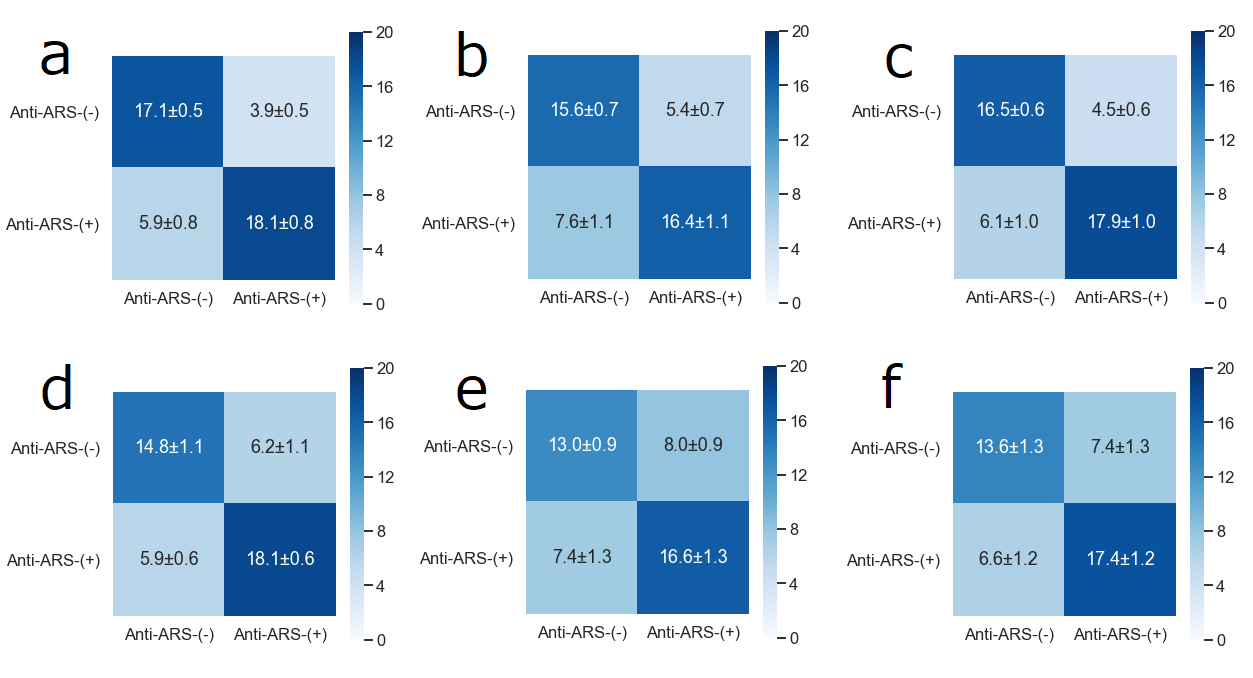
Supplementary Figure S14. Confusion matrices show the status of binary classifications for anti-ARS autoantibodies with a linear discriminant analysis (LDA) classifier (**a**), quadratic discriminant analysis (QDA) classifier (**b**), support vector machine (SVM) classifier (**c**), k-nearest neighbors (k-NN) classifier (**d**), random forest (RF) classifier (**e**), and multi-layer perceptron (MLP) classifier (**f**). Data are means ± standard deviations.


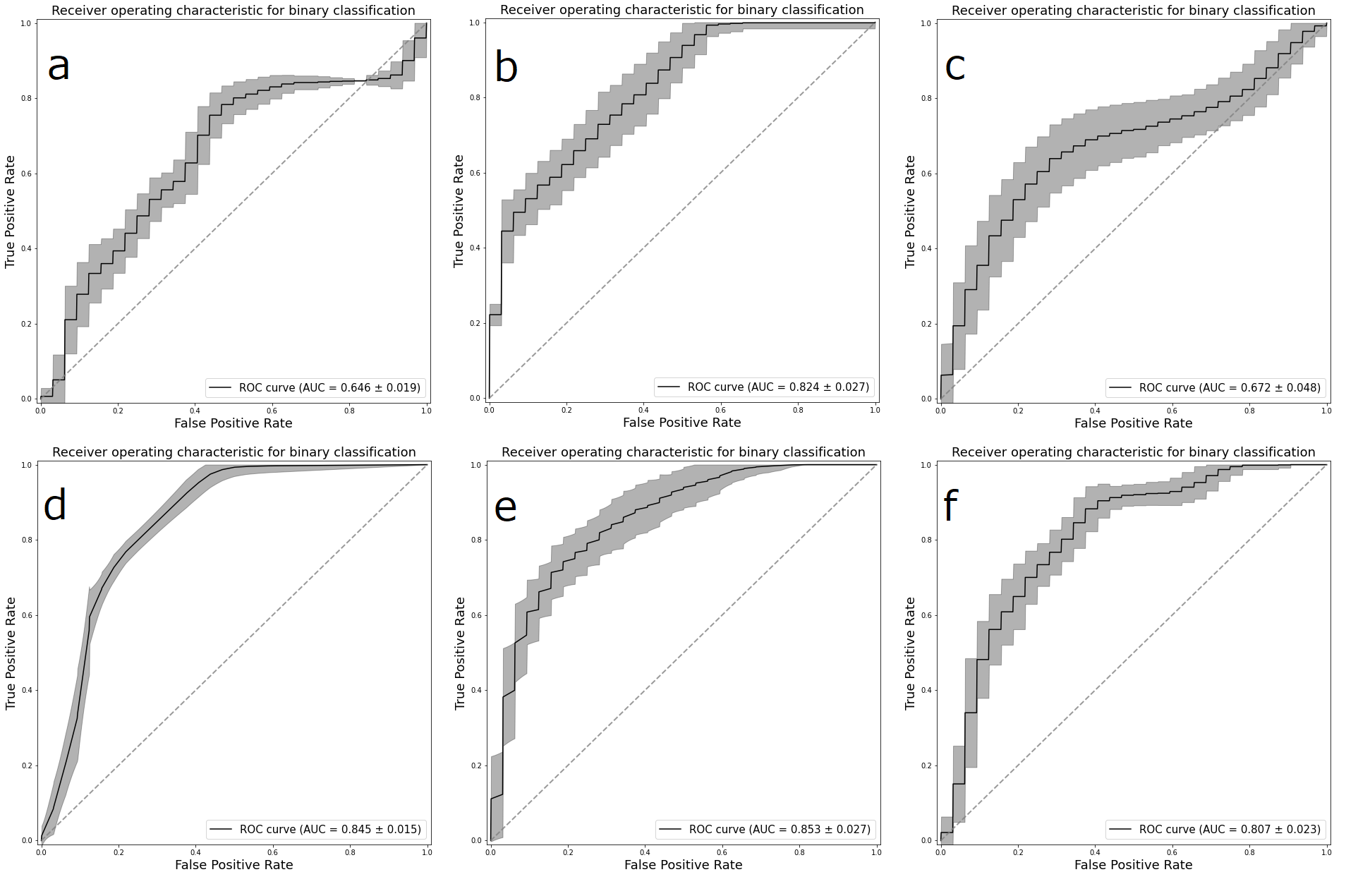


Supplementary Figure S15. ROC curves and AUC values of binary classifications for anti-Jo-1 autoantibodies with a linear discriminant analysis (LDA) classifier (**a**), quadratic discriminant analysis (QDA) classifier (**b**), support vector machine (SVM) classifier (**c**), k-nearest neighbors (k-NN) classifier (**d**), random forest (RF) classifier (**e**), and multi-layer perceptron (MLP) classifier (**f**). AUCs are means ± standard deviations.


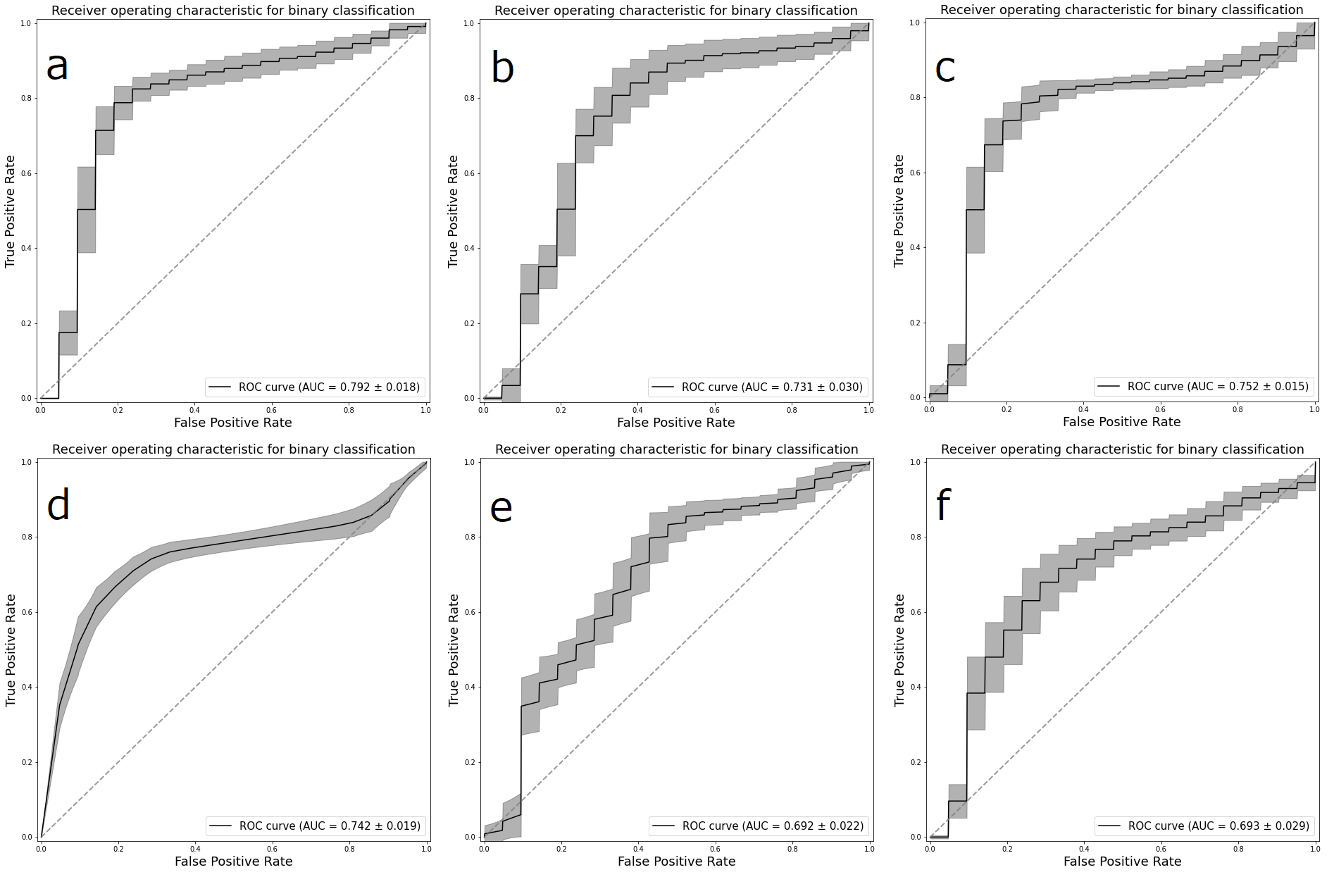


Supplementary Figure S8. ROC curves and AUC values of binary classifications for anti-ARS autoantibodies with a linear discriminant analysis (LDA) classifier (**a**), quadratic discriminant analysis (QDA) classifier (**b**), support vector machine (SVM) classifier (**c**), k-nearest neighbors (k-NN) classifier (**d**), random forest (RF) classifier (**e**), and multi-layer perceptron (MLP) classifier (**f**). AUCs are means ± standard deviations.

Supplementary Table S1. Demographic and clinical characteristics of study patients

Disease subtype classification (n=74)

PM DM ADM Non-IIM

(n=19) (n=22) (n=14) (n=19) p<0.05

Age (years)^b^, 63.4 ± 9.7 56.8 ± 14.1 48.9 ± 11.9 58.1 ± 10.9 PM vs ADM

mean ± SD non-IIM vs ADM

Female^a^ 13 (68.4%) 12 (54.5%) 10 (71.4%) 13 (68.4%) DM vs ADM

Muscle weakness*^a^ 18 (94.7%) 20 (90.9%) 4 (28.6%) 13 (72.2%) PM vs ADM

DM vs ADM

Non-IIM vs PM

Non-IIM vs DM

Non-IIM vs ADM

CK level (IU/L)^b^, 2911.8 3728.0 468.2 1115.3 PM vs ADM

mean ± SD ± 3292.2 ± 5127.2 ± 653.7 ± 977.1 DM vs ADM

Non-IIM vs PM

Non-IIM vs DM

Non-IIM vs ADM

Biopsy performed

Muscle** 16 (84.2%) 15 (68.2%) 4 (28.6%) 12 (66.7%) N/A

Skin*** 2 (10.5%) 21 (95.5%) 14 (100%) 8 (42.1%) N/A

Note—Except where otherwise indicated, data are the number (%) of patients. N/A, not applicable; SD, standard deviation.

a Data were tested using the chi-squared test.

b Data were tested using the *t*-test.

*Objective symmetric weakness of the proximal upper extremities, objective symmetric weakness of the proximal lower extremities, or weakness of the neck flexors (weaker than neck extensors).

**Myositis was confirmed on muscle histopathology in 16 patients with PM and 14 with DM. Muscle weakness in the proximal extremities or neck flexor muscles and markedly elevated serum CK levels were confirmed in the 3 other patients with PM and 8 with DM.

***Dermal changes consistent with DM/ADM were confirmed in 18 patients with DM and 11 with ADM. In 4 other patients with DM and 3 with ADM, skin manifestations (Gottron’s sign/Heliotrope rash) were confirmed by dermatologists.

Table S2. Performance of classifications of non-IIM and IIM groups (original features)

Accuracy Recall Precision AUC

(%) (%) (%)

LDA macro-average 42.7±2.6 41.2±2.6 40.3±3.5 0.627±0.013

(TexF1+TexF8 Non-IIM 14.5±5.8 27.0±9.9 0.403±0.037

+TexF9+TexF10) PM 51.3±5.6 44.0±4.3 0.660±0.017

DM 65.2±3.6 46.8±2.0 0.729±0.016

ADM 33.9±4.4 43.4±4.7 0.677±0.017

QDA macro-average 39.1±2.4 36.9±2.3 37.5±2.7 0.633±0.014

(TexF1+TexF3 Non-IIM 43.1±5.2 40.0±4.6 0.649±0.021

+TexF7+TexF11) PM 28.5±4.8 30.2±4.6 0.523±0.026

DM 58.8±4.7 45.9±3.5 0.692±0.024

ADM 17.4±4.1 33.8±6.8 0.630±0.034

SVM macro-average 34.4±2.7 31.4±2.5 26.0±2.8 0.492±0.031

(TexF2+TexF8 Non-IIM 34.3±6.5 28.4±4.8 0.432±0.062

+TexF10+TexF11) PM 37.4±4.2 33.2±3.9 0.465±0.053

DM 53.6±5.0 41.1±3.1 0.540±0.054

ADM 0.2±1.2 1.2±7.2 0.495±0.050

k-NN macro-average 37.0±3.0 34.3±2.7 29.4±2.4 0.556±0.015

(TexF4+TexF5 Non-IIM 60.3±5.7 35.9±3.2 0.616±0.035

+TexF8+TexF10) PM 35.4±6.6 32.5±5.0 0.548±0.033

DM 41.6±5.8 49.1±5.5 0.682±0.020

ADM 0.0±0.0 0.0±0.0 0.378±0.035

RF macro-average 36.3±2.7 35.1±2.6 35.2±2.7 0.627±0.018

(TexF1+TexF2 Non-IIM 32.6±6.4 33.3±5.5 0.612±0.033

+TexF3+TexF11) PM 26.2±5.9 27.2±5.5 0.510±0.037

DM 52.9±5.9 47.6±4.6 0.709±0.025

ADM 28.8±4.7 32.7±5.6 0.669±0.027

MLP macro-average 42.6±2.7 40.9±2.6 42.8±3.2 0.628±0.012

(TexF1+TexF4 Non-IIM 32.0±5.8 35.8±5.5 0.546±0.027

+TexF6+TexF9) PM 46.1±5.7 44.0±4.8 0.615±0.024

DM 59.0±4.5 44.8±2.7 0.711±0.017

ADM 26.5±4.4 46.6±7.7 0.604±0.023

Note— Data are means ± standard deviations. Feature name codes are as follows: TexF1 = kurtosis, TexF2 = interquartile range, TexF3 = total energy, TexF4 = cluster prominence, TexF5 = correlation, TexF6 = difference average, TexF7 = imc2, TexF8 = maximum probability, TexF9 = large dependence high gray-level emphasis, TexF10 = dependence non-uniformity, TexF11 = coarseness. LDA = linear discriminant analysis, QDA = quadratic discriminant analysis, SVM = support vector machine, k-NN = k-nearest neighbors classifier, RF = random forest classifier, MLP = multi-layer perceptron.

Table S3. Performance of classifications of non-IIM and IIM groups (LBP features)

Accuracy Recall Precision AUC

(%) (%) (%)

LDA macro-average 41.3±2.5 40.2±2.4 41.5±2.8 0.618±0.013

(TexFL1+TexFL5 Non-IIM 32.3±5.6 35.2±5.9 0.478±0.033

+TexFL6+TexFL8) PM 29.8±6.6 27.6±4.6 0.571±0.025

DM 61.9±2.9 53.4±3.1 0.701±0.016

ADM 36.7±3.0 49.6±5.7 0.684±0.021

QDA macro-average 36.3±2.6 36.9±2.7 36.6±2.7 0.612±0.014

(TexFL1+TexFL6 Non-IIM 34.4±4.7 31.1±4.0 0.539±0.025

+TexFL8+TexFL9) PM 30.3±4.8 35.1±4.9 0.578±0.027

DM 38.0±4.5 41.5±4.6 0.557±0.027

ADM 44.7±7.8 38.5±5.5 0.739±0.024

SVM macro-average 39.1±2.9 38.7±3.0 40.5±3.6 0.628±0.019

(TexFL2+TexFL3 Non-IIM 22.5±7.3 22.0±6.5 0.378±0.050

+TexFL6+TexFL8) PM 45.9±6.3 40.4±4.0 0.593±0.048

DM 47.8±3.6 45.8±3.0 0.709±0.020

ADM 38.5±6.0 53.7±8.1 0.792±0.016

k-NN macro-average 36.6±2.8 35.0±2.7 38.5±3.4 0.655±0.016

(TexFL1+TexFL2 Non-IIM 33.1±6.4 26.6±4.1 0.570±0.030

+TexFL6+TexFL9) PM 34.7±6.4 32.6±4.9 0.671±0.024

DM 51.6±5.1 47.3±4.3 0.675±0.023

ADM 20.5±3.6 47.3±8.8 0.704±0.030

RF macro-average 43.2±3.3 42.5±3.3 45.6±4.1 0.657±0.016

(TexFL1+TexFL4 Non-IIM 35.8±8.9 35.4±6.9 0.608±0.046

+TexFL5+TexFL7) PM 36.5±6.1 38.6±5.3 0.583±0.030

DM 57.0±4.5 45.8±4.1 0.693±0.022

ADM 40.7±5.5 62.6±10.2 0.737±0.026

MLP macro-average 38.3±3.0 38.3±3.0 39.2±3.4 0.663±0.014

(TexFL3+TexFL4 Non-IIM 33.1±5.4 35.7±5.0 0.560±0.025

+TexFL6+TexFL7) PM 40.1±5.0 42.0±5.0 0.664±0.022

DM 40.7±5.9 35.0±3.8 0.610±0.025

ADM 39.5±5.8 44.2±7.0 0.781±0.020

Note— Data are means ± standard deviations. Feature name codes are as follows: TexFL1 = total energy, TexFL2 = variance, TexFL3 = cluster shade, TexFL4 = contrast, TexFL5 = difference entropy, TexFL6 = long run emphasis, TexFL7 = long run low gray-level emphasis, TexFL8 = gray-level non-uniformity, TexFL9 = busyness. LDA = linear discriminant analysis, QDA = quadratic discriminant analysis, SVM = support vector machine, k-NN = k-nearest neighbors classifier, RF = random forest classifier, MLP = multi-layer perceptron.

Table S4. Information on the representative MSA/MAA status in IIM and non-IIM patients

Antibodies

ARS Jo-1 n Comments

Non-IIM (+) (+) 1 1 with anti-Ro-52-(+)

(n=19) (+) (-) 3 2 with anti-PL-7-(+) and anti-Ro-52-(+)

(-) (-) 7 3 with anti-SRP-(+), 1 with anti-TIF1-γ-(+)

and 3 with anti-Ro-52-(+)

N/D (-) 8 1 with anti-PM-Scl-75-(+) and anti-U1RNP-(+)

PM (+) (+) 4 1 with anti-Ro-52-(+)

(n=19) (+) (-) 3 3 with anti-PL-7-(+) and anti-Ro-52-(+)

(-) (-) 7 3 with anti-SRP-(+), 1 with anti-U1RNP-(+),

2 with anti-Ro-52-(+) and 1 with anti-Ku-(+)

N/D (-) 5 1 with anti-Ku-(+)

DM (+) (+) 5 1 with anti-Ro-52-(+)

(n=22) (+) (-) 4 2 with anti-EJ-(+) , 2 with anti-PL-12-(+)

and 2 with anti-Ro-52-(+)

(-) (-) 3 3 with anti-TIF1-γ-(+)

N/D (-) 10 3 with anti-Ro-52-(+) and 1 with anti-Ku-(+)

ADM (+) (+) 3

(n=14) (+) (-) 1 1 with anti-Ro-52-(+)

(-) (-) 4 4 with anti-MDA5-(+) and 2 with anti-Ro-52-(+)

N/D (-) 6 1 with anti-Ro-52-(+)

Note— n=number of patients, N/D=no data.

The representative source codes for multi-class classification analysis :


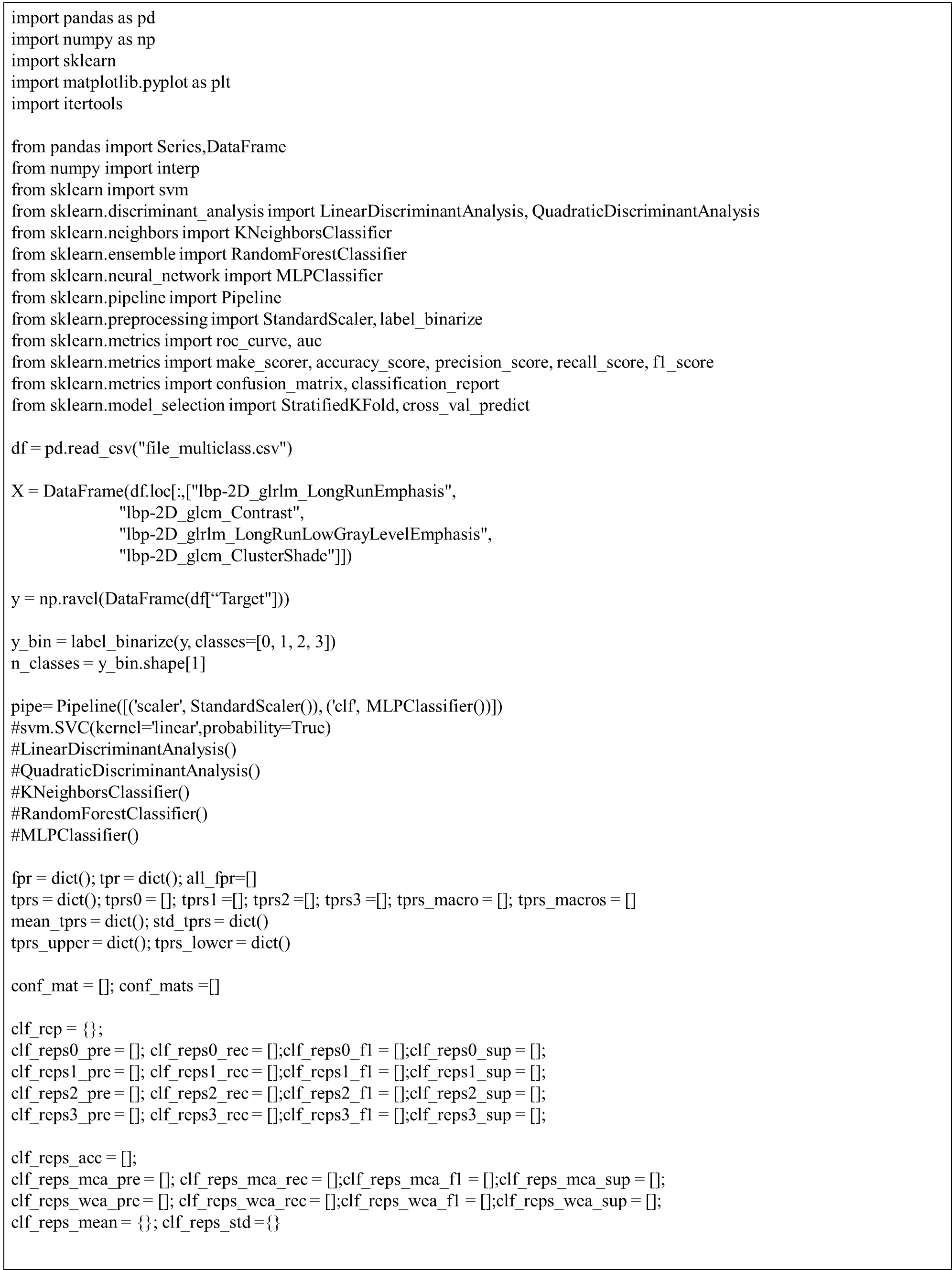


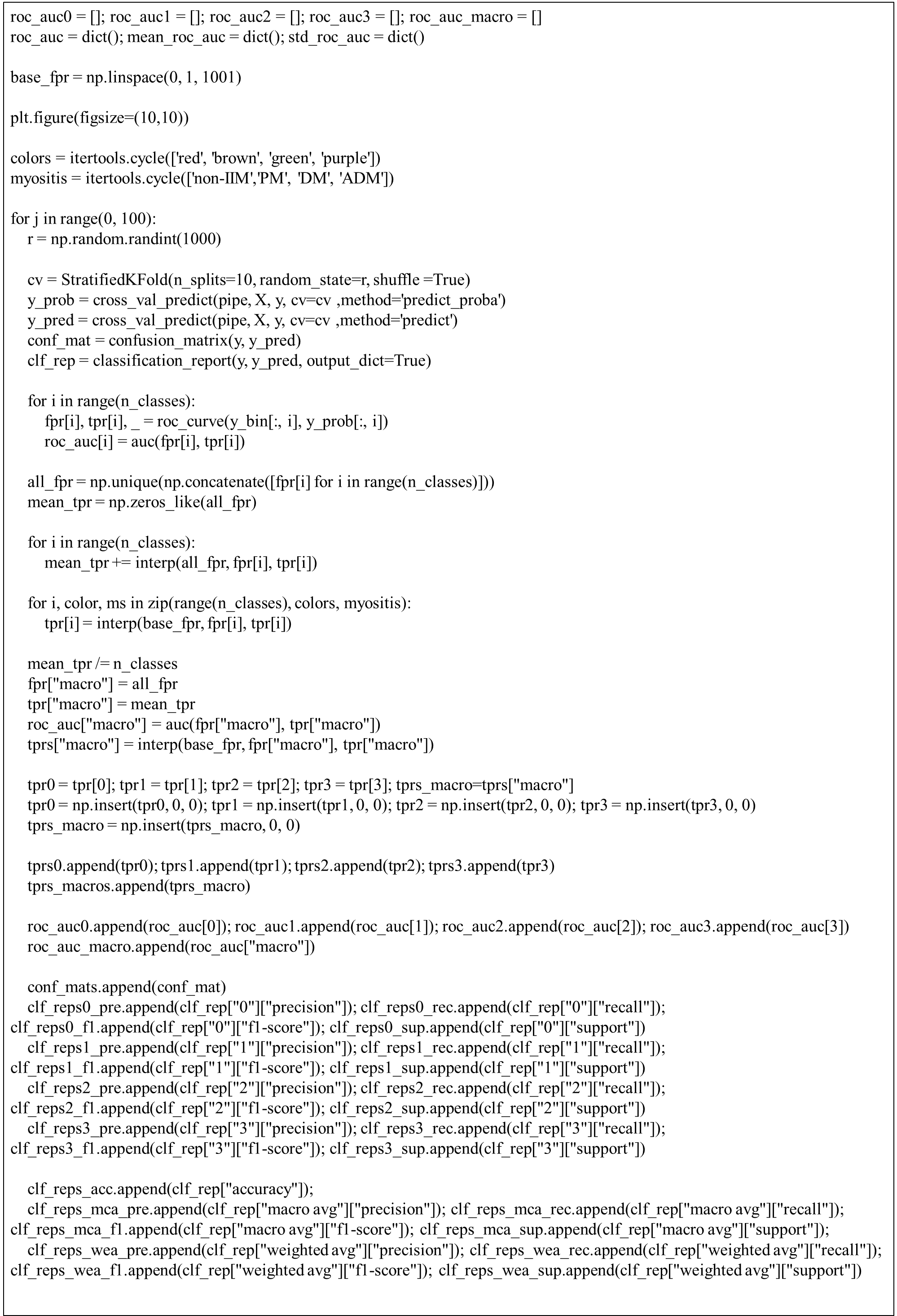


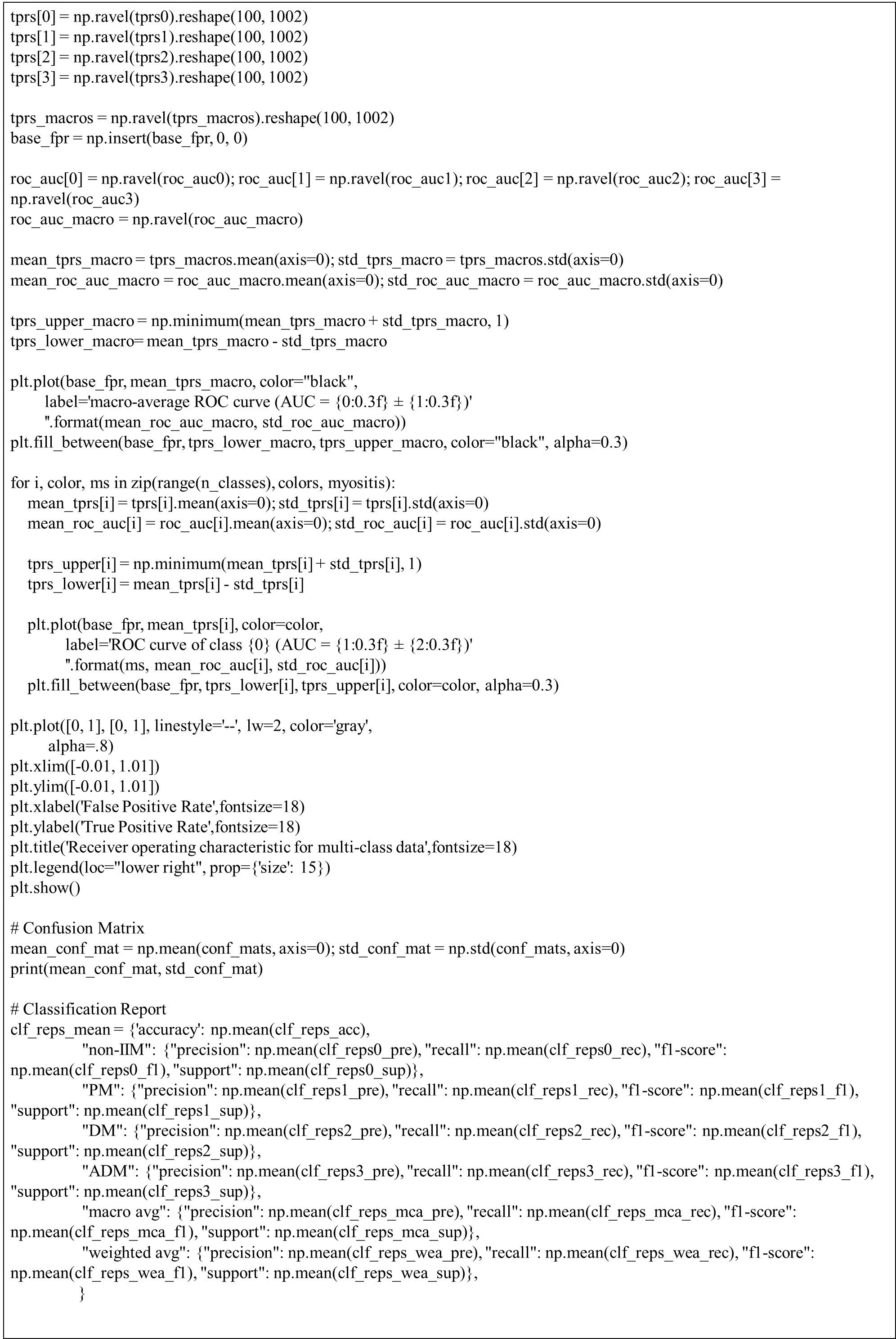


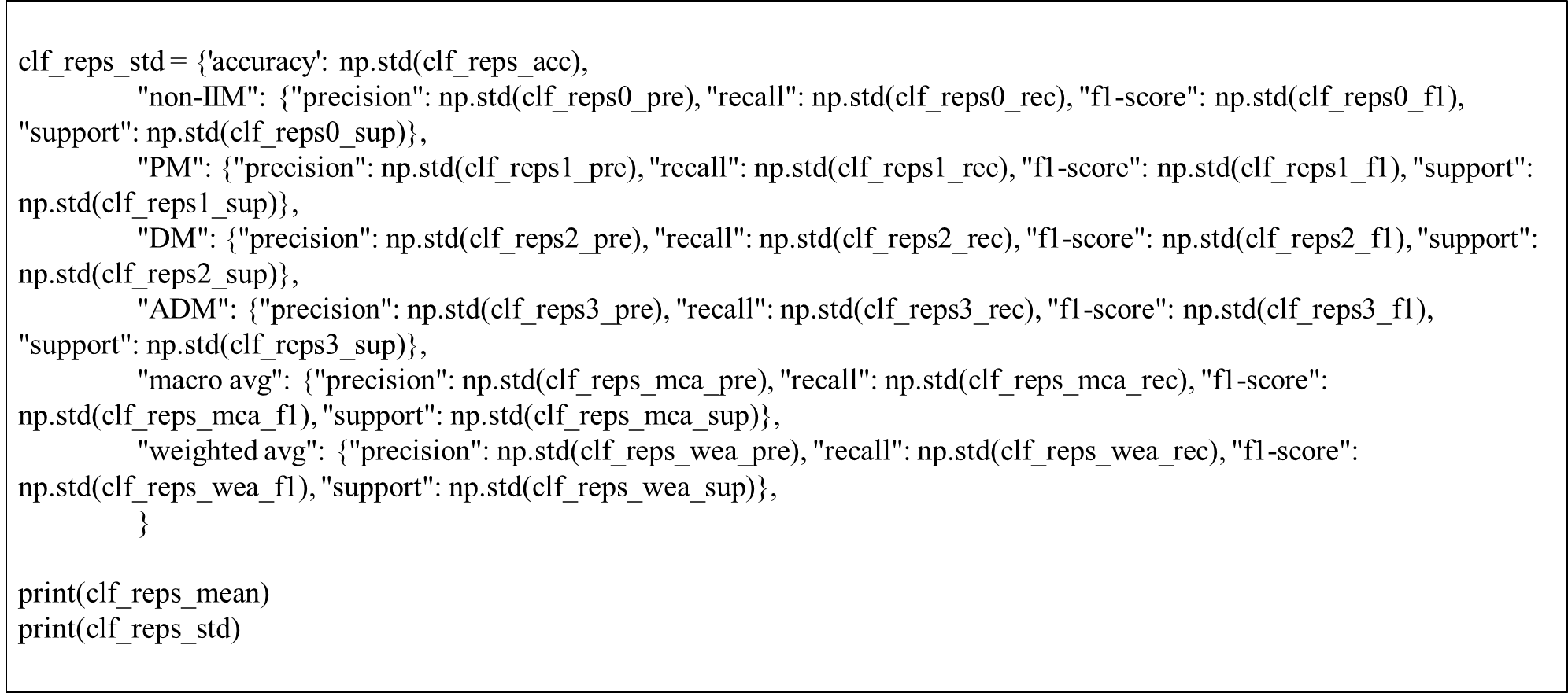


The representative source codes for binary classification analysis :


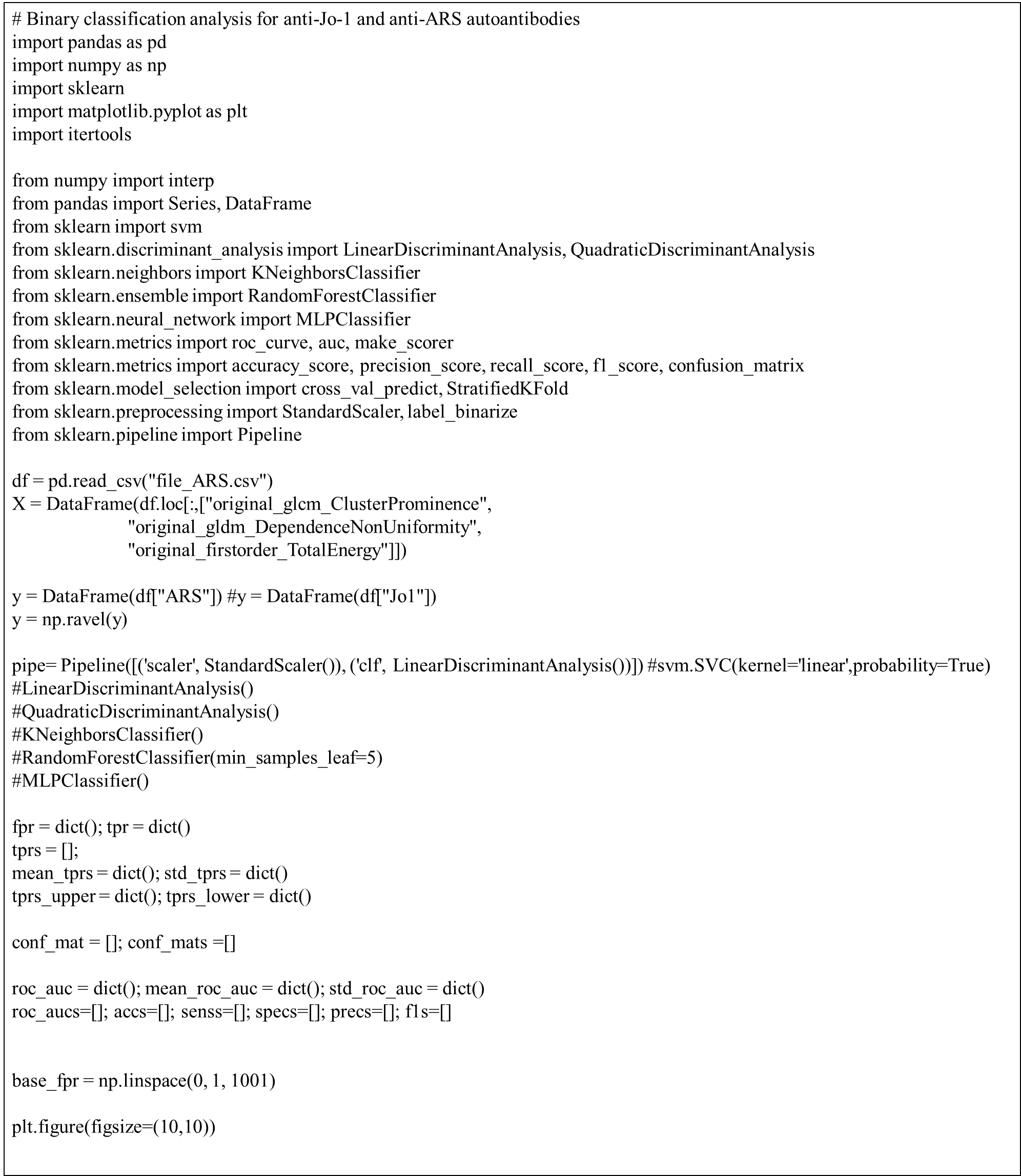


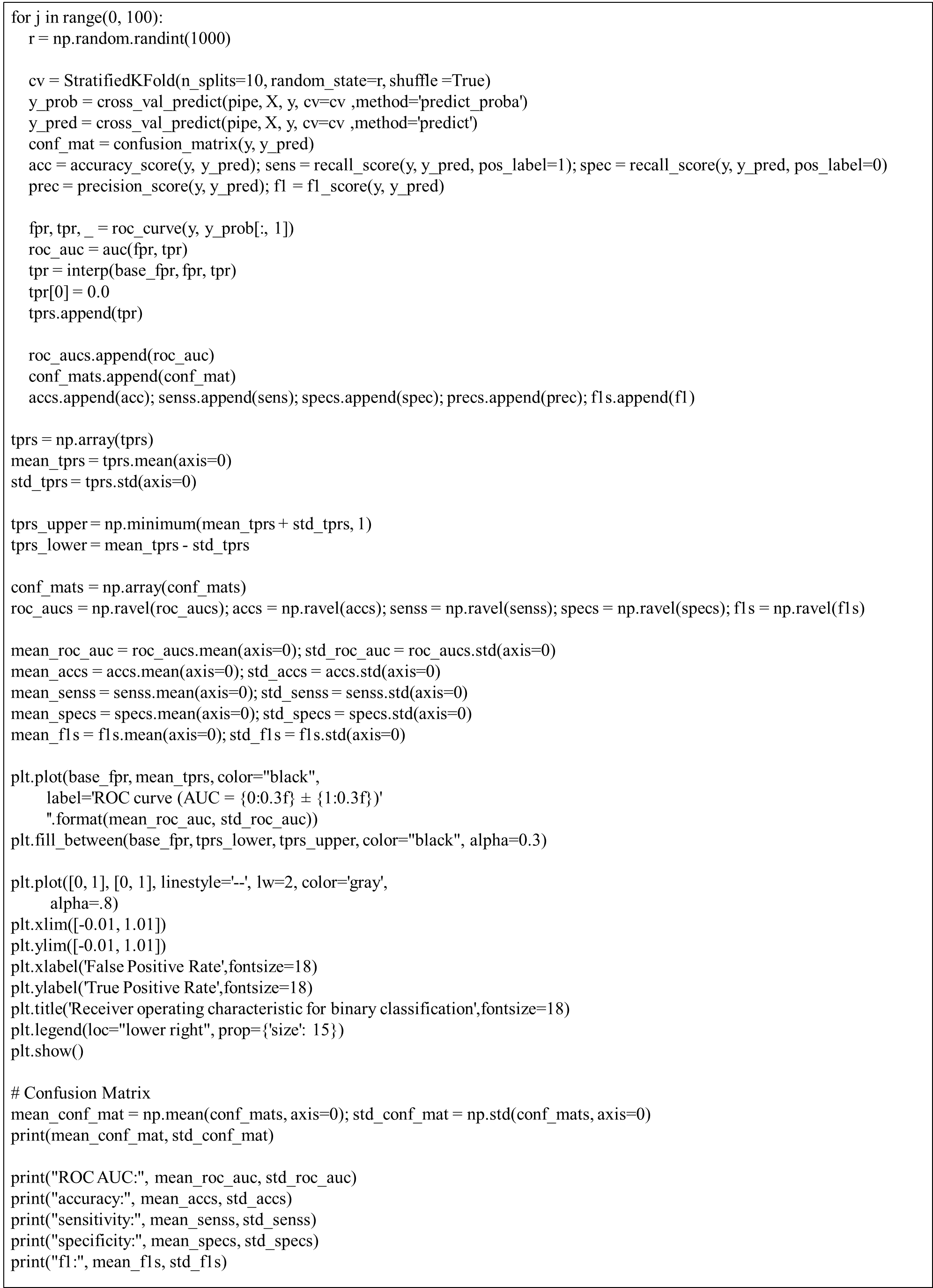

Supplement: Supplementary file 1 — Supplementary Information. [file 41598_2021_89311_MOESM1_ESM.docx]
